# Genomic Insights of “*Candidatus* Nitrosocaldaceae” Based on Nine New Metagenome-Assembled Genomes, Including “*Candidatus* Nitrosothermus” Gen Nov. and Two New Species of “*Candidatus* Nitrosocaldus”

**DOI:** 10.3389/fmicb.2020.608832

**Published:** 2021-01-08

**Authors:** Zhen-Hao Luo, Manik Prabhu Narsing Rao, Hao Chen, Zheng-Shuang Hua, Qi Li, Brian P. Hedlund, Zhou-Yan Dong, Bing-Bing Liu, Shu-Xian Guo, Wen-Sheng Shu, Wen-Jun Li

**Affiliations:** ^1^State Key Laboratory of Biocontrol, Guangdong Provincial Key Laboratory of Plant Resources and Southern Marine Science and Engineering Guangdong Laboratory (Zhuhai), School of Life Sciences, Sun Yat-sen University, Guangzhou, China; ^2^Department of Biological Sciences, Dartmouth College, Hanover, NH, United States; ^3^School of Life Sciences, University of Nevada, Las Vegas, Las Vegas, NV, United States; ^4^Nevada Institute of Personalized Medicine, University of Nevada, Las Vegas, Las Vegas, NV, United States; ^5^Henan Key Laboratory of Industrial Microbial Resources and Fermentation Technology, College of Biological and Chemical Engineering, Nanyang Institute of Technology, Nanyang, China; ^6^School of Life Sciences, South China Normal University, Guangzhou, China

**Keywords:** “*Candidatus* Nitrosocaldaceae”, hot spring, ammonia-oxidizing archaea, genome comparison, horizontal gene transfer

## Abstract

“*Candidatus* Nitrosocaldaceae” are globally distributed in neutral or slightly alkaline hot springs and geothermally heated soils. Despite their essential role in the nitrogen cycle in high-temperature ecosystems, they remain poorly understood because they have never been isolated in pure culture, and very few genomes are available. In the present study, a metagenomics approach was employed to obtain “*Ca.* Nitrosocaldaceae” metagenomic-assembled genomes (MAGs) from hot spring samples collected from India and China. Phylogenomic analysis placed these MAGs within “*Ca.* Nitrosocaldaceae.” Average nucleotide identity and average amino acid identity analysis suggested the new MAGs represent two novel species of “*Candidatus* Nitrosocaldus” and a novel genus, herein proposed as “*Candidatus* Nitrosothermus.” Key genes responsible for chemolithotrophic ammonia oxidation and a thaumarchaeal 3HP/4HB cycle were detected in all MAGs. Furthermore, genes coding for urea degradation were only present in “*Ca.* Nitrosocaldus,” while biosynthesis of the vitamins, biotin, cobalamin, and riboflavin were detected in almost all MAGs. Comparison of “*Ca*. Nitrosocaldales/Nitrosocaldaceae” with other AOA revealed 526 specific orthogroups. This included genes related to thermal adaptation (cyclic 2,3-diphosphoglycerate, and S-adenosylmethionine decarboxylase), indicating their importance for life at high temperature. In addition, these MAGs acquired genes from members from archaea (Crenarchaeota) and bacteria (Firmicutes), mainly involved in metabolism and stress responses, which might play a role to allow this group to adapt to thermal habitats.

## Introduction

Geothermal springs represent a model system to study microbial ecology because of their simple community structure and simplified biogeochemical processes (Amend and Shock, 2001). This habitat serves as an important niche for diverse archaea with heterogeneous metabolic capabilities (Alves et al., 2018). Among the various modes of chemolithotrophy, ammonia oxidation can be a major energy source in some geothermal systems, due to relatively high concentrations of ammonia (Dodsworth et al., 2012). The global distribution of archaeal ammonia monooxygenase subunit A (*amoA*) in terrestrial geothermal springs (Zhang et al., 2008), and high rates of nitrification in some hot springs (Reigstad et al., 2008; Dodsworth et al., 2011), suggests a vital role for thermophilic ammonia-oxidizing archaea (AOA) in the nitrogen and carbon cycles in these habitats. “*Candidatus* Nitrosocaldus” members are the only archaea capable of oxidizing ammonia to nitrite at temperatures >65°C (de la Torre et al., 2008; Abby et al., 2018; Daebeler et al., 2018). Members of this family are globally distributed in neutral or slightly alkaline hot spring sediments or geothermally heated soils (Marteinsson et al., 2001; Nunoura et al., 2005; de la Torre et al., 2008; Zhang et al., 2008; Dodsworth et al., 2011; Hou et al., 2013; Hamilton et al., 2014; Abby et al., 2018).

“*Candidatus* Nitrosocaldaceae,” which includes “*Ca*. Nitrosocaldus,” was first proposed in 2008 (de la Torre et al., 2008) to include the first known member of the genus, “*Ca.* N. yellowstonensis.” So far, only three enrichment cultures of “*Ca.* Nitrosocaldus” have been reported: “*Ca.* N. yellowstonensis” from a Yellowstone National Park hot spring, “*Ca.* N. islandicus” from an Icelandic hot spring, and “*Ca.* N. cavascurensis” SCU2 from a hot spring in southern Italy (de la Torre et al., 2008; Abby et al., 2018; Daebeler et al., 2018). Like other AOA, all of them are chemolithoautotrophic and couple ammonia oxidation to carbon fixation using the 3-hydroxypropionate/4-hydroxybutyrate pathway (de la Torre et al., 2008; Abby et al., 2018; Daebeler et al., 2018). Besides ammonia oxidation, additional metabolic activities have been suggested from these three genomes, including aromatic amino acid fermentation, utilization of urea, nitrile, and hydrogen cyanide as alternative ammonia donors, and synthesis of vitamins (de la Torre et al., 2008; Abby et al., 2018; Daebeler et al., 2018). Despite these critical features, the family remains poorly understood because of the lack of pure cultures, limited availability of genomes, and small number of studies measuring their activities *in situ*.

In the present study, a metagenomics approach was employed to obtain “*Ca.* Nitrosocaldaceae” metagenomic-assembled genomes (MAGs) from hot spring samples collected from India and China. With the newly recovered MAGs and available AOA genomes, we have performed comparative genomic analyses to provide insight into the phylogeny, functional diversity, and adaptation mechanisms of this family and propose one novel genus and two novel species.

## Materials and Methods

### Sampling, DNA Extraction, Sequencing, and Phylogenetic Analysis

Hot spring sediment samples were collected from India (Gujarat, Tumba: 22°47′58″N 73°27′37″E, Temp: 55°C) and China (Yunnan, Jinze, JZ: 25°26′28″N 98°27’36″E, Temp: 75°C; Qiaoquan, QQ: 24°57′0″N 98°26′11″E, Temp: 69°C; Shuirebaozha, SRBZ: 24°57′0″N 98°26’14″E, Temp: 72°C). General descriptions for the Chinese hot springs have been described elsewhere (Hedlund et al., 2012; Hou et al., 2013).

Genomic DNA was extracted using the PowerSoil DNA isolation kit (MoBio). Metagenomic sequencing data were generated using the Illumina HiSeq 4000 instrument. The raw data were processed as described by Hua et al. (2015) and assembled using SPAdes v.3.14.0 (Bankevich et al., 2012). The sequence coverage was determined by BBMap v.36.77^1^ and genome binning based on tetra-nucleotide frequencies, and sequencing depth was conducted with MetaBAT v.2.12.1 (Kang et al., 2015). The quality of the MAGs was evaluated using CheckM v.1.0.7 (Parks et al., 2015). Full-length 16S rRNA gene sequences were extracted from the MAGs using rnammer v.1.2 (Lagesen et al., 2007). “*Ca.* Nitrosocaldaceae” MAGs were preliminarily screened using PhyloPhlAn v.0.99 (Segata et al., 2013). Phylogenetic tree was constructed using a concatenated set of 122 universal marker proteins by GTDB-Tk (Parks et al., 2017). MUSCLE v.3.8.31 (Edgar, 2004) with default parameters used to align these sequences. Poorly aligned regions were removed using TrimalAL v.1.4 (Capella-Gutiérrez et al., 2009). The phylogeny was inferred with IQtree v.1.6.0 (Nguyen et al., 2014) by ultra-rapid bootstraps (1,000) with the parameters –alrt 1000 –bb 1000 –nt AUTO.” Besides, nucleotide sequences of *amoA* gene were retrieved (Alves et al., 2018) and aligned with MAFFT v.7.407 (Katoh and Standley, 2013). Protein sequences coding for nitrite reductase (NirK), multicopper oxidases (Decleyre et al., 2016; Kerou et al., 2016) and indolepyruvate ferredoxin oxidoreductase (Daebeler et al., 2018) were retrieved and aligned with MAFFT. The alignments were filtered using TrimalAL v.1.4 (Capella-Gutiérrez et al., 2009) to eliminate columns with ≥ 95% gaps, and all gene trees were inferred with IQtree v.1.6.0 (Nguyen et al., 2014). All trees were uploaded to iTOL v.4 (Letunic and Bork, 2019) for visualization.

### Genome Annotation, Horizontal Gene Transfer Predictions, and Comparative Genomics

In order to gain a comprehensive insight into “*Ca.* Nitrosocaldaceae,” published “*Ca.* Nitrosocaldaceae” MAGs were obtained from NCBI^2^ and JGI^3^. Protein-coding regions were identified using Prodigal v.2.6.3 (Hyatt et al., 2010) with the “-p single” option and annotated against the Kyoto Encyclopedia of Genes and Genomes (KEGG) (Kanehisa et al., 2016) and Archaeal Clusters of Orthologous Genes (arCOG) (Makarova et al., 2015) database (e-value threshold = 10^–5^) with DIAMOND (Buchfink et al., 2015). Assignments of key metabolic pathways and specific functions were manually verified based on the KEGG result and the online KEGG mapping tools^4^. Horizontal gene transfers (HGTs) were inferred using HGTector2 (Zhu et al., 2014) and visualized using SankeyMATIC^5^. For confirmation, we use candidate HGT protein sequences selected in our MAGs as queries against the protein sequences of NCBI genomes database available (downloaded in May 2020). Retrieved protein sequences together with protein sequences in “*Ca.* Nitrosocaldaceae” were then aligned using MAFFT, the alignments were filtered with TrimalAL and trees were constructed with IQtree and visualized in iTOL. All parameter sets were the same as mentioned above. For simplicity, the initial phylogenetic trees were used to choose a small set of sequences in the final trees. The rRNAs and tRNAs were predicted using RNAmmer v.1.2 (Lagesen et al., 2007) and tRNAscan-SE v.2.0.2, respectively (Lowe and Eddy, 1997). The average nucleotide identity (ANIm) values of MAGs were determined using pyani (Pritchard et al., 2016), and the average amino acid identity (AAI) values were determined using CompareM^6^. For further comparative analysis, MAGs with completeness ≥80% were selected. OrthoFinder v.2.4.0 (parameters “-s blast_gz –t 20 –f protein_files”) with BLASTP (-e 0.001) was used to assess orthology among the coding sequences (CDS) of all selected genomes (Emms and Kelly, 2015, 2019). The term “shared set” was defined for orthogroups that were present in a given branch and other branches, whereas the “specific set” refers to orthogroups that were only present in a given branch. Then, further sub-defining the “specific set,” the term “lineage-core set” was used for orthogroups identified in ≥90% of the analyzed genomes, and the term “lineage-accessory set” was used for orthogroups that were present in <90% of the analyzed genomes. Detailed information on published AOA used in this study is provided in Supplementary Table S1.

## Results and Discussion

### General Features of “*Ca.* Nitrosocaldaceae” MAGs

In total, nine “*Ca.* Nitrosocaldaceae” MAGs were obtained from Chinese and Indian hot spring sediments. Among them, four MAGs were obtained from Jinze (JZ-1.bins.77, JZ-2.bins.172, JZ-2.bins.249, and JZ-3.bins.102), three from Qiaoquan (QQ.bins.88, QQ.bins.97, and QQ.bins.115137), one from Shuirebaozha (SRBZ.bins.174), and one from Tumba (Tumba.bins.72).

**FIGURE 1:**
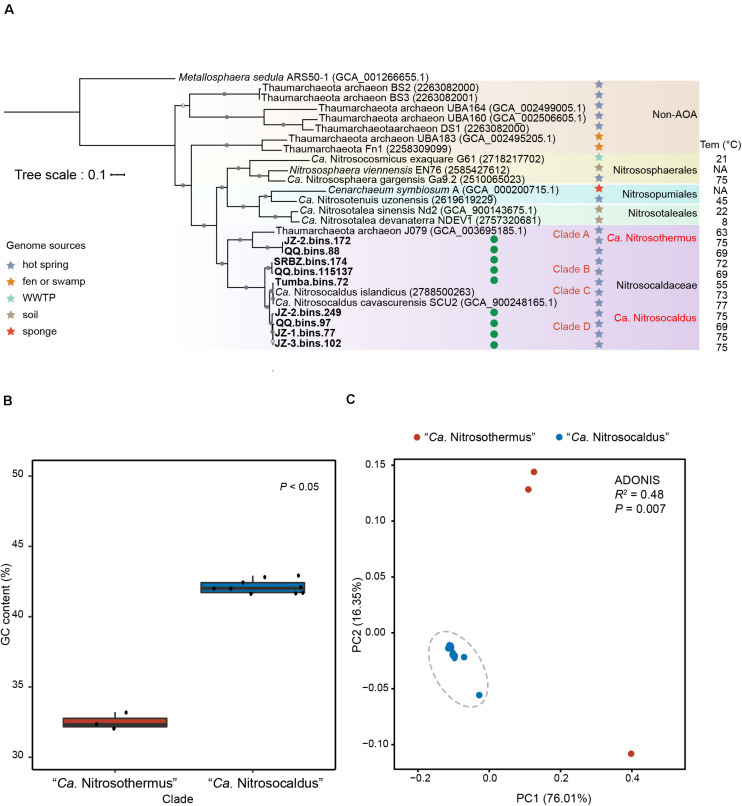
**(A)** Phylogenetic tree of Thaumarchaeota based on concatenation of 122 archaeal marker genes with *Metallosphaera sedula* ARS50-1 as outgroup (only a few genomes of other Thaumarchaeota are shown). MAGs newly obtained in this study are marked with green circles. Sources of Group HWCG-III MAGs are indicated by stars with different colors. The temperature values refer to temperature measured in the source materials. Nodes with ultrafast bootstrap value ≥95% (50%) are indicated as solid (hollow) circles, and the scale bar at the top indicates 10% sequence divergence. **(B)** Genome G+C content of Clade A (“*Ca*. Nitrosothermus”) vs. Clades B, C, and D (“*Ca*. Nitrosocaldus”). **(C)** Principal coordinates analysis (PCoA) plot based on Bray-Curtis dissimilatory of KEGG functional profiles of “*Ca.* Nitrosocaldaceae” MAGs, showing significantly different KEGG profiles in Clade A (“*Ca*. Nitrosothermus”) vs. Clades B, C, and D (“*Ca*. Nitrosocaldus”).

In addition to the two cultivated members of the genus “*Ca.* Nitrosocaldus” (with genomic data), “*Ca.* N. cavascurensis SCU2” (Abby et al., 2018) and “*Ca.* N. islandicus” (Daebeler et al., 2018), a MAG (Thaumarchaeota archaeon J079) obtained from a Japanese hot spring (Ward et al., 2019) was also assigned to this family based on phylogenetic analysis. In phylogenetic trees based on alignments of 122 archaeal marker genes (Figure 1A), a monophyletic group containing the two cultivated members of “*Ca*. Nitrosocaldus” and the nine additional MAGs were recovered.

Phylogenetic analysis (Figure 1A) showed that the twelve MAGs formed four well-supported clades within the family “*Ca.* Nitrosocaldaceae.” Clade A consisted of JZ-2.bins.172, QQ.bins.88, and Thaumarchaeota archaeon J079. Clade B corresponded to QQ.bins.115137 and SRBZ.bins.174. Clade C included Tumba.bins.72 and two recently proposed “*Ca.* Nitrosocaldus” species, “*Ca.* N. islandicus” and “*Ca.* N. cavascurensis SCU2.” Clade D comprised of JZ-2.bins.249, JZ-3.bins.102, JZ-1.bins.77, and QQ.bins.97.

AAI values suggested that clades B, C, and D can be ascribed to the genus “*Ca*. Nitrosocaldus,” as their AAI values (Supplementary Table S2 and Supplementary Figure S1A) were within the proposed limits (65–95%) (Konstantinidis et al., 2017). In contrast, AAI values comparing Clade A genomes to “*Ca*. Nitrosocaldus” genomes were well below those values (56.6–57.8%). In addition, other genomic characteristics consistently separated Clade A from “*Ca*. Nitrosocaldus,” reflected by GC contents (Clade A, 31.9–33.1%, average 32.4%, *n* = 3; “*Ca.* Nitrosocaldus,” 41.5–42.8%, average 42.0%, *n* = 9, Figure 1B) and overall KO profiles (Figure 1C, ADONIS *R*^2^ = 0.48, P < 0.01).

Therefore, we propose the name “*Candidatus* Nitrosothermus koennekii” gen. nov., sp. nov. to circumscribe the organisms represented by clade A.

Etymology: Ni.tro.so.ther’mus. L. masc. adj. *nitrosus*, full of natron, here intended to mean nitrous; Gr. masc. adj. *thermos*, hot; N.L. masc. n. *Nitrosothermus*, a nitrite-forming organism from a hot environment; koen.ne’ke.i. N.L. gen. n. *koennekei*, named in honor of Martin Könneke. The type genome is JZ-2.bins.172^T^.

The ANI values (Supplementary Table S3 and Supplementary Figure S1B) separating clades B, C, and D were below the cut-off (95–96%) for species identification (Jain et al., 2018), and we propose the names “*Candidatus* Nitrosocaldus tengchongensis” sp. nov. and “*Candidatus* Nitrosocaldus schleperae” sp. nov. for clade B and clade D, respectively. The type genomes are QQ.bins.115137^T^ for “*Candidatus* Nitrosocaldus tengchongensis” and JZ-1.bins.77^T^ for “*Candidatus* Nitrosocaldus schleperae.”

Etymology: teng.chong.en’sis. N.L. masc. adj. *tengchongensis* pertaining to Tengchong, Yunnan Province, south-west China, where the type strain was isolated; schle’pe.rae. N.L. gen. n. *schleperae*, named in honor of Christa Schleper.

Despite the wide geographic range of clade C (Italy, Iceland, and India) and the previous descriptions of two “*Ca.* Nitrosocaldus” species, “*Ca.* N. cavascurensis” (Abby et al., 2018) and “*Ca.* N. islandicus” (Daebeler et al., 2018), the high ANI values between members of clade C (98.41–99.80%) (Supplementary Table S3) suggests clade C as a single species group.

The estimated genome sizes were small (1.26–1.56 Mb), with estimated completeness of 58.8–100% and estimated contamination of 0–3.4%. Most MAGs have detectable 16S rRNA genes (except Thaumarchaeota archaeon J079) and more than 18 tRNAs (except JZ-2.bin.249), indicating that they were well curated and appropriate for further analysis. The number of coding sequences ranged from 857 to 1,767, with about half of them being assignable by KO profiles. The detailed features of these MAGs are given in Table 1.

**TABLE 1:** Genomic features of “*Candidatus* Nitrosocaldaceae”.

Genome feature	1	2	3	4	5	6	7	8	9	10	11	12
No. of scaffolds	434	38	34	1	1	12	56	196	111	88	16	26
Genome size (Mbp)	1.06	1.51	1.39	1.50	1.54	1.50	1.18	1.04	0.63	1.39	1.39	1.39
Estimated Genome Size (Mbp)	1.26	1.51	1.43	1.52	1.56	1.52	1.29	1.40	1.08	1.41	1.40	1.41
Genomic G+C content (%)	33.1	32.2	31.9	41.6	41.5	41.5	42.8	42.7	42.3	41.9	42	41.9
*N*_50_ value (bp)	3,037	1,56,292	1,85,164	15,77,284	16,17,394	2,23,702	29,463	6,454	6,672	29,350	1,23,923	1,02,733
No. of protein coding genes	1,569	1,767	1,648	1,718	1,753	1,729	1,431	1,457	857	1,700	1,665	1,646
No. of rRNAs	1	2	2	2	2	2	2	2	1	2	2	2
Presence of 16S rRNA gene	No	Yes	Yes	Yes	Yes	Yes	Yes	Yes	Yes	Yes	Yes	Yes
No. of tRNAs	32	40	37	36	36	41	31	30	14	36	38	39
No. of genes annotated by KO	811 (51.7%)	877 (49.6%)	819 (49.7%)	832 (48.4%)	836 (47.6%)	840 (48.5%)	746 (52.1%)	751 (51.5%)	488 (56.9%)	849 (49.9%)	818 (49.1%)	812 (49.3%)
Completeness^*a*^ (%)	84.43	100	97.5	99	99	99	91.9	74.7	58.8	98	99	98
Contamination^*a*^ (%)	2.5	0.9	0	0	0	0.9	0	0	3.4	0	0	0
Accession number	GCA_ 003695185.1^*b*^	GCA_ 013538795.1^*b*^	GCA_ 013538715.1^*b*^	GCA_ 900248165.1^*b*^	2788500263^*c*^	GCA_ 011058825.1^*b*^	GCA_ 013538675.1^*b*^	GCA_ 013538755.1^*b*^	GCA_ 013538775.1^*b*^	GCA_ 013538695.1^*b*^	GCA_ 013538805.1^*b*^	GCA_ 013538705.1^*b*^

**1, Thaumarchaeota archaeon J079; 2, JZ-2.bins.172; 3, QQ.bins.88; 4, “Ca. Nitrosocaldus cavascurensis”; 5, “Ca. Nitrosocaldus islandicus”; 6, Tumba.bins.72; 7, QQ.bins.115137; 8, SRBZ.bins.174; 9, JZ-2.bins.249, 10, JZ-3.bins.102; 11, JZ-1.bins.77; 12, QQ.bins.97. ^*a*^The evaluations of genome completeness and contamination were conducted with CheckM. Although the genomes of “Ca. Nitrosocaldus islandicus” and “Ca. Nitrosocaldus cavascurensis” are only 99% complete in the table, these are, in fact, closed genomes that are missing PF000395. ^*b*^The GenBank accession number. ^*c*^The JGI accession number.**

### Metabolic Potential of “*Ca.* Nitrosocaldaceae”

#### Nitrogen Metabolism

**FIGURE 2:**
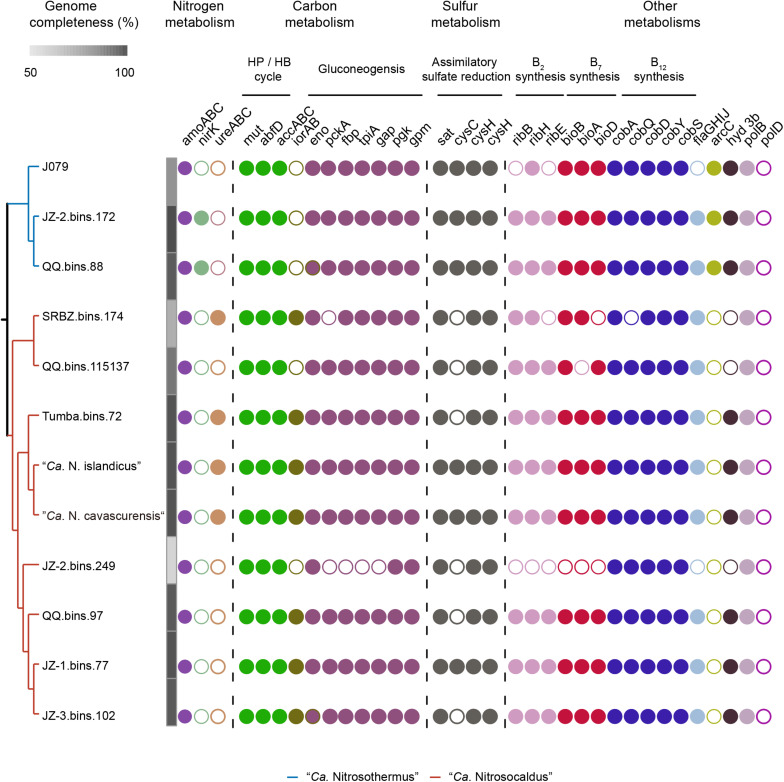
The overall distributions of the genes of interest in “*Ca.* Nitrosocaldaceae.” The phylogenetic tree in the left was pruned according to Figure 1. For visualization, the branch length is ignored. The two genera were colored according to Figure 1. MAGs of “*Ca.* Nitrosothermus” are marked with blue while MAGs of “*Ca*. Nitrosocaldus” with red. The solid and hollow circles represent the presence and absence of the genes. J079 Thaumarchaeota archaeon J079, HP/HB cycle hydroxypropionate/hydroxybutyrate cycle, *amoABC* ammonia monooxygenase subunit A, B, and C, *nirK* nitrite reductase, *ureABC* urease subunit gamma, beta and alpha, *mut* cobalamin-dependent methylmalonyl-CoA mutase, *abfD* 4-hydroxybutyryl-CoA dehydratase, *accABC* biotin-dependent acetyl-CoA/propionyl-CoA carboxylase, *iorAB* indolepyruvate ferredoxin oxidoreductase subunit alpha and beta, *pckA* phosphoenolpyruvate carboxykinase, *fbp* fructose 1,6-bisphosphate aldolase/phosphatase, *tpiA* triosephosphate isomerase, *gap*, glyceraldehyde-3-phosphate dehydrogenase, *pgk* phosphoglycerate kinase, *gpm* phosphoglycerate mutase, *eno* enolase, *sat* sulfate adenylyltransferase, *cysC* adenylylsulfate kinase, *cysH* phosphoadenosine phosphosulfate reductase, sir sulfite reductase (ferredoxin), *flaGHIJ* archaeal flagellar protein FlaG, FlaH, FlaI, and FlaH, *arcC* carbamate kinase, hyd 3b group 3b hydrogenase, *polB* archaea type DNA polymerase, *polD* D-family polymerase. The full name of genes involved in B_2_ (riboflavin), B_7_ (biotin), and B_12_ (cobalamin) syntheses are listed in Supplementary Table S4.

Similar to other AOA (Walker et al., 2010; Spang et al., 2012), the key genes responsible for ammonia oxidation encoded by *amoABC* were present in all “*Ca.* Nitrosocaldaceae” MAGs (Figure 2, Supplementary Figure S2, and Supplementary Table S4).

**FIGURE 3:**
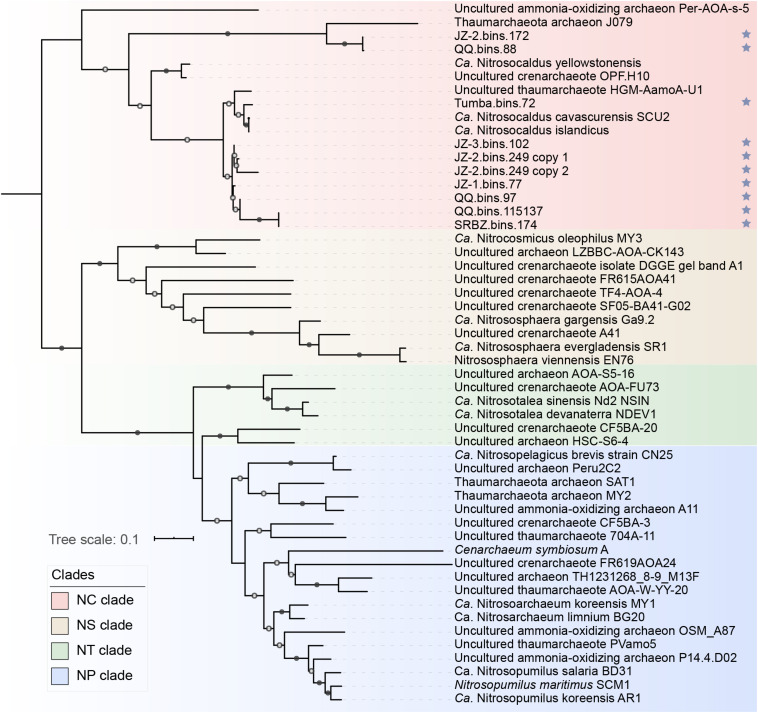
Phylogenetic analysis of *amoA* gene sequences. Sequences with blue stars were newly obtained in this study. Classifications of clades were based on Alves et al. (2018). Only nodes with ultrafast bootstrap values ≥95% (50%) were marked with solid (hollow) circles, and the scale bar at the bottom indicates 10% sequence divergence.

Ammonia oxidation not only yields free energy but also reducing force for anabolic reactions. Previously, an additional copy of *amoC* that may participate in an ammonia starvation and stress response was observed in some AOA MAGs (Berube et al., 2007; Berube and Stahl, 2012; Spang et al., 2012). In our study, an extra *amoC* could be found in two “*Ca.* Nitrosothermus” MAGs (Supplementary Table S4), indicating that they may grow at a lower concentration of ammonia than other “*Ca.* Nitrosocaldaceae” members. Interestingly, a full-length extra *amoA* gene (NODE_52239_length_4113_cov_3.91646_6) was detected in MAG JZ-2.bins.249. The sequence assembly of the extra *amoA*-containing scaffold (NODE_52239_length_4113_cov_3.91646) seems to be reliable, as the GC content and read depth of this scaffold was similar to other scaffolds in this bin (Supplementary Figure S3). All the closest protein sequences of this scaffold showed high similarities to “*Ca.* Nitrosocaldus cavascurensis” (Supplementary Table S5). However, the additional *amoA* gene was near the end of the scaffold and the gene content and orientation between the two scaffolds were highly identical. Besides, the extra *amoA* genes was found in the most contaminated MAG (contamination: 3.4%) but these two copies of *amoA* genes were very closely related (100% in query coverage and 99% in amino acid identity, Figure 3). We conclude that the placement of the second *amoA* gene copy into the MAG might be due to strain diversity, which lead to incorrect assignment of two very similar contigs into the same MAG. Therefore, we assume that the species represented by the MAG were likely to harbor only one *amoA* gene. In addition, all *amoA* genes in “*Ca.* Nitrosocaldaceae” were placed in the NC-clade (Figure 3), which only occupies a small fraction of all known *amoA* sequences (Alves et al., 2018), stressing the limited research on this family.

It is well-known that ammonia can be imported into the cell via passive diffusion (Winkler, 2006). The detection of an AMT family ammonia transporter in most MAGs makes it possible to actively transport ammonia into cells when the permeability of the cytoplasmic membrane becomes low, or the concentrations of ammonia were relatively low, as passive diffusion would be limited in these conditions (Winkler, 2006). It has been reported that multiple copper oxidases may play some roles in hydroxylamine oxidation, the second step of ammonia oxidation (Walker et al., 2010). In our study, genes encoding multicopper oxidases (MCO) were identified in some MAGs (Supplementary Table S4), which indicates that alternative mechanisms may exist for hydroxylamine oxidation. It has been suggested that NirK may provide NO during the NO-dependent dehydrogenation of hydroxylamine to nitrite (Kozlowski et al., 2016).

**FIGURE 4:**
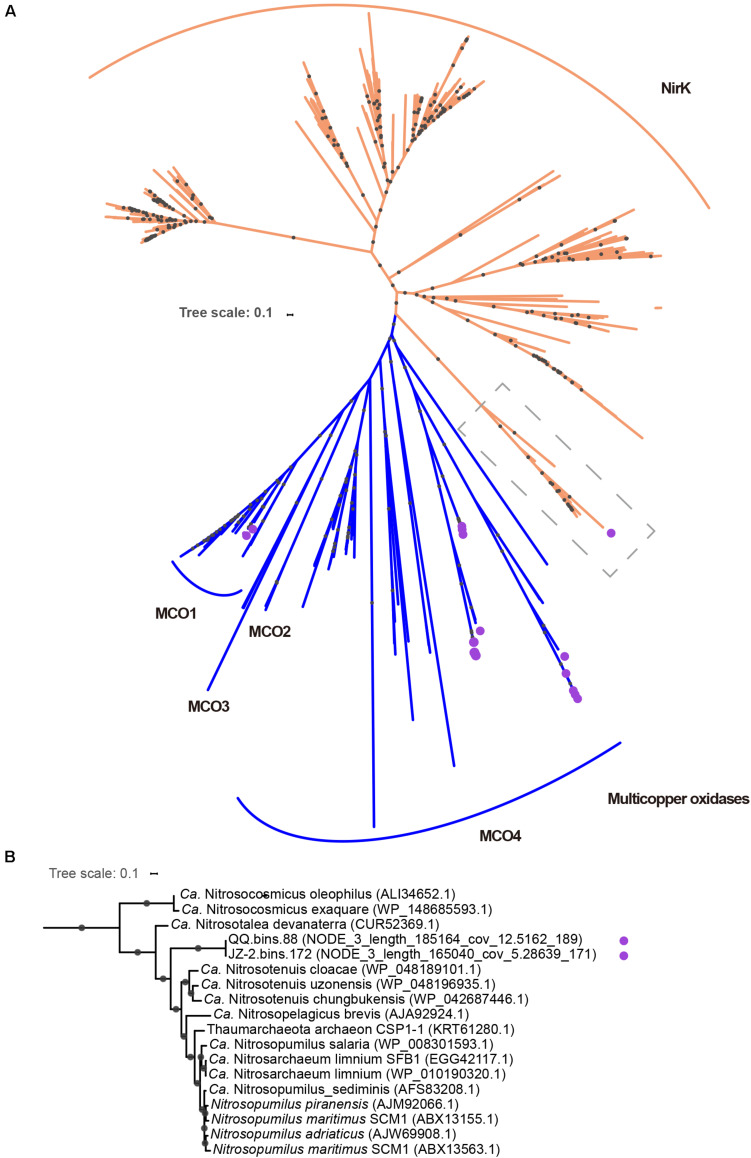
**(A)** The full unrooted tree for NirK and multicopper oxidases (MCOs) included sequences retrieved from our study (with purple circles), references from Decleyre et al. (2016) and Kerou et al. (2016). The full tree was constructed from an alignment of 406 sequences. Sequences encoded by *nirK* gene are indicated in orange, and multicopper oxidase genes in blue. Dashed boxes in gray contained *nirK* gene identified in our study (“*Ca.* Nitrosocladaceae”) and from other Thaumarchaeota genomes. Clades of MCOs were based on phylogenetic trees in Kerou et al. (2016). **(B)** The expanded version of the subsection in the dashed box. Nodes with ultrafast boot strap ≥95% (50%) are indicated as solid (hollow) circles and the scale bar indicates 10% sequence divergence.

Homologs for NirK were found in two MAGs (JZ-2.bins.172 and QQ.bins.88). Interestingly, these MAGs harbor two copies of this protein (Supplementary Table S4). The phylogenetic analysis revealed that only one NirK (NODE_3_length_185164_cov_12.5162_189 in QQ.bins.88 and NODE_3_length_165040_cov_5.28639 in JZ-2.bins.172) in each MAG was placed adjacent to the previously defined archaeal NirK branch (Figure 4; Kerou et al., 2016). An alignment with these archaeal NirK proteins showed that type-1 and type-2 copper centers were conserved in these two newly recovered proteins (Supplementary Figure S4). Consistent with the previous study, they encode a potential transmembrane domain with a signal peptide at N-terminus (Bartossek et al., 2010). After careful phylogenetic analysis, we claim that these two proteins were genuine copper-dependent nitrite reductases with functions similar to characterized NirK instead of homologous MCO. The other homologous proteins clustered with multicopper oxidase sequences (Figure 4A), indicating they likely encode multicopper oxidases instead of NirK. Six MCOs belong to lineage 1 (MCO1, Figure 4A and Supplementary Table S6), which were from four “*Ca.* Nitrosocaldus” MAGs and two “*Ca*. Nitrosothermus” MAGs, while twenty-six MCOs belong to lineage 4 (MCO4, Figure 4A and Supplementary Table S6) were sourced from all MAGs. Notably, in one MAG (QQ.bins.115137), one ZIP family permease was detected next to MCO1. This pair of genes plays a role in copper sequestration (Kerou et al., 2016). It was assumed that MCO1 and MCO4 were involved in Cu uptake as they could carry out the oxidation of Cu^+^ to Cu^2+^, which could be transported into the cells via the ZIP family permease or a divalent transporter (Reyes et al., 2020). MCO could aid “*Ca.* Nitrosocaldaceae” not only in ammonia oxidation but also copper homeostasis.

Urea degradation provides AOA with ammonia to fuel ammonia oxidation and carbon dioxide for carbon fixation (Walker et al., 2010; Spang et al., 2012). In our study, only four MAGs belonging to “*Ca.* Nitrosocaldus” possess genes coding for urease and urea transporters, which suggests that among “*Ca.* Nitrosocaldaceae” urea utilization was unique in this genus. Consistent with a previous report (Daebeler et al., 2018), all members in “*Ca.* Nitrosocaldaceae” can potentially utilize hydrogen cyanide and nitrile, which were available in geothermal systems (Miller and Urey, 1959; Schulte and Shock, 1995). Nitrilases catalyze the degradation of nitrile, and cyanide hydratases convert HCN to formamide, and both can produce ammonia (Lenza and Vasconcelos, 2001; Pace and Brenner, 2001) to support ammonia oxidation. The capability to utilize additional nitrogen-containing substrates could provide an advantage when ammonia was limited. The presence of *glnA* and *gdnA* genes coding for glutamine synthetase and glutamate dehydrogenase suggests that “*Ca.* Nitrosocaldaceae” could generate glutamate via ammonia assimilation.

#### Carbon Metabolism

The key enzymes of the thaumarchaeal 3HP/4HB cycle were detected in all MAGs, indicating the potential for carbon fixation and the most energy-efficient pathway that could generate acetyl-CoA for biosynthesis (Könneke et al., 2014). The detection of an incomplete Embden-Meyerhof-Parnas pathway (EMP), genes coding for phosphoenolpyruvate carboxykinase, and fructose 1,6-bisphosphate aldolase in most MAGs support gluconeogenesis and sugar production (Figure 2, Supplementary Figure S2, and Supplementary Table S4). Except for some low-completeness MAGs, members of this family harbor an oxidative TCA cycle, which could support free-energy yielding reactions with organic substrates and/or provide some important intermediates for biosynthesis (e.g., oxaloacetic acid and α-ketoglutaric acid). Additionally, genes coding for 4-oxalocrotonate tautomerase, essential for the metabolism of aromatic compounds, was detected in all MAGs, indicating that they could generate intermediates for the TCA cycle via conversion of aromatic compounds. The detection of the pentose phosphate pathway and 5-phospho-alpha-D-ribose 1-diphosphate PRPP biosynthesis in this group suggests they can generate some nucleotide and amino acid precursors.

Furthermore, “*Ca.* Nitrosocaldus” might have the potential for aromatic amino acid fermentation due to the presence of *iorAB*, encoding for indolepyruvate ferredoxin oxidoreductase, which was not common among AOA (Daebeler et al., 2018).

Inferred from the phylogenetic tree (IorB protein sequences), they form a monophyletic clade, which was placed between bacterial IorB proteins, and a Crenarchaeota-Euryarchaeota cluster (Supplementary Figure S5). Besides, the presence of hydrogenases could regenerate oxidized ferredoxin that reduced during this process using hydrogen as an energy source, as proposed before (Daebeler et al., 2018). Furthermore, several enzymes, including aspartate aminotransferase argininosuccinate synthase and argininosuccinate lyase, were detected in “*Ca.* Nitrosocaldaceae” MAGs, suggesting a potential anaplerotic contribution of amino acids to the carbon and energy flow. Moreover, genes *pepP* and *pepN*, coding for aminopeptidases, were found in most of the MAGs together with the ABC-type peptidase/nickel transporter system and *iorAB*, suggesting that members of “*Ca.* Nitrosocaldaceae” could assimilate amino acids for energy or intermediates for biosynthesis.

Another enzyme detected in Thaumarchaeota archaeon J079 was carbonic anhydrase, catalyzing the conversion between CO_2_ and bicarbonate, indicating that it could provide this MAG with bicarbonate, the substrate for the HP/HB cycle (Kerou et al., 2016). The capability of carbon fixation combined with the detection of various pathways involved in organic carbon metabolism suggests metabolic versatility of “*Ca.* Nitrosocaldaceae” as reported for other Thaumarchaeota (Tourna et al., 2011; Lehtovirta-Morley et al., 2014), providing a hint for mixotrophy in “*Ca.* Nitrosocaldaceae.” However, these metabolisms require verification.

#### Oxidative Phosphorylation

The aerobic lifestyle of “*Ca.* Nitrosocaldus” has previously been reported in enrichment cultures (de la Torre et al., 2008; Abby et al., 2018; Daebeler et al., 2018). Except for one “*Ca.* Nitrosothermus” MAG (Thaumarchaeota archaeon J079), the universal presence of cytochrome c-type terminal oxidase (Complex III) in “*Ca.* Nitrosocaldaceae” MAGs confirmed that they could use oxygen as a terminal electron acceptor. Additionally, Complex I (NADH: ubiquinone oxidoreductase), II (succinate: quinone oxidoreductase), IV (cytochrome c oxidase), and V (ATPase) of the respiratory chain were detected in all MAGs.

#### Other Metabolisms

Several vitamins, such as biotin, cobalamin, and riboflavin were essential cofactors that were required for different enzymes (Berg et al., 2007; Mansoorabadi et al., 2007; Kim and Winge, 2013; Chow et al., 2018), including biotin-dependent acetyl-CoA/propionyl-CoA carboxylase and cobalamin-dependent muthylmalony-CoA mutase, which were part of the HP/HB cycle (Ishii et al., 1996). Key enzymes involved in the biosynthesis of these cofactors were identified in most of the “*Ca.* Nitrosocaldaceae” MAGs (Figure 2, Supplementary Figure S2, and Supplementary Table S4). Homologs of these genes were also reported in some other Thaumarchaeota lineages (Doxey et al., 2015; Santoro et al., 2015; Heal et al., 2018). The only exception was JZ-2_bins.249, whose completeness was only 58.8%, so we attributed the lack of these genes in this MAG due to genome incompleteness. The genetic capacity for *de novo* synthesis of these cofactors was conserved in “*Ca.* Nitrosocaldaceae,” indicating that cofactor synthesis could be an important service for terrestrial microbial communities, as these cofactors could be synthesized by some but not all prokaryotes (Zhang et al., 2009; Swithers et al., 2012). Other genes (*sat*, *cysH*, and *sir*) involved in assimilatory sulfate reduction were found in these MAGs, indicating they can conduct this pathway. The products could be used for amino acid synthesis. In contrast, other “*Ca.* Nitrosocaldaceae” MAGs cannot reduce sulfate in this way as they lack CysC, which was responsible for converting adenylyl sulfate to 3’-phosphoadenylyl sulfate. Motility has been reported in some AOA (Lehtovirta-Morley et al., 2016). In our study, most MAGs encode genes responsible for archaeal flagellar proteins (*flaG*, *flaH*, *flaI*, and *flaJ*). The only exceptions were Thaumarchaeota archaeon J079 and JZ-2.bins.249, which has low completeness (84.4 and 58.8%). Therefore, motility may be a common feature of “*Ca.* Nitrosocaldaceae,” which could be advantageous for responding to environmental conditions (Blainey et al., 2011). Although phosphate transporter-related genes were commonly found, alkaline phosphatase was only found in “*Ca.* Nitrosothermus” MAGs, which might confer an advantage in phosphorus-limiting environments (Shen et al., 2016). Moreover, *arcC*, coding for carbamate kinase and *carB*, coding for carbamoyl-phosphate synthase were only detected in “*Ca.* Nitrosothermus” (Supplementary Table S4). These two enzymes could potentially provide ammonia under aerobic conditions (Abdelal et al., 1982), which could be valuable given the low concentration of ammonia in these springs (Hedlund et al., 2012; Hou et al., 2013; Ward et al., 2019) and many other alkaline geothermal springs (Holloway et al., 2011). However, the potential function of these genes in ammonia production in “*Ca.* Nitrosothermus” deserves more attention.

### Comparison Between “*Ca.* Nitrosocaldales/Nitrosocaldaceae” and Other AOA

To gain insight into potential mechanisms enabling thermophily in “*Ca.* Nitrosocaldales/Nitrosocaldaceae,” comparative genomics of all selected AOA was conducted.

**FIGURE 5:**
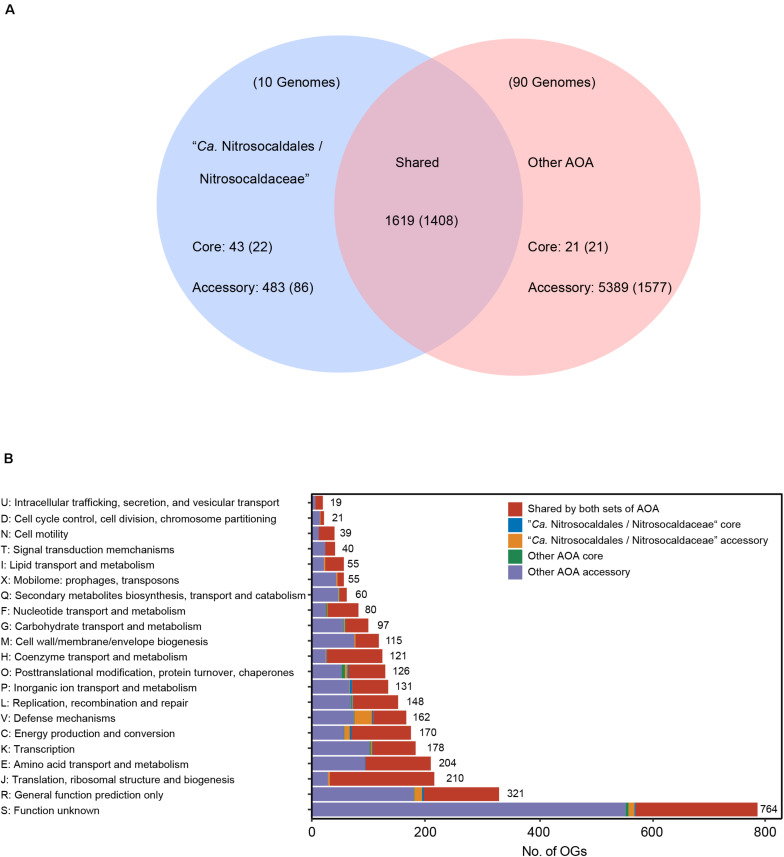
**(A)** Venn diagram illustrating the number of OGs shared between “*Ca*. Nitrosocaldales/Nitrosocaldaceae” and other AOA, and the number of OGs only present in either set. Numbers in parentheses indicate the number of OGs that could be assigned to arCOG database. **(B)** Distribution of the arCOG functional categories assigned to the defined sets. Detailed information is provided in Supplementary Table S7.

The term “shared set” was defined for orthogroups that were present in a given branch and other branches, whereas the “specific set” refers to orthogroups that were only present in a given branch. Then, further sub-defining the “specific set,” the term “lineage-core set” was used for orthogroups identified in ≥90% of the analyzed genomes, and the term “lineage-accessory set” was used for orthogroups that were present in <90% of the analyzed genomes. A total of 179,946 coding sequences (CDS) of all selected AOA MAGs were clustered into 15,802 OGs, with 8,247 classified as singletons. Comparisons of OGs between “*Ca.* Nitrosocaldaceae” and other AOA enabled us to identify a shared set of 1,619 protein families, and a large number of these OGs could be assigned to information-processing genes (replication, transcription, translation) (Figure 5 and Supplementary Table S7). Among them, all genes of the highly conserved central information-processing machinery in Thaumarchaeota were detected (Spang et al., 2010), including ribosomal proteins (S25, S26, S30, L13e, L29), other proteins involved in translation (RNA polymerase subunit B, transcription factor MBF1), topoisomerases IB, proteins involved in cell division (Cell division ATPase of the AAA^+^ class ESCRT system component C, cell division GTPase FtsZ, chromatin segregation and condensation protein ScpA and ScpB), histones H3 and H4, and proteins involved in the repair of macromolecules (ERCC4-like helicase, ERCC4-type nuclease, chaperone DnaK, molecular chaperon GrpE). As discussed above, we have detected genes participating in the thaumarchaeal 3HP/4HB pathway, TCA cycle, gluconeogenesis, non-oxidative PPP, ammonia oxidation, and biosynthesis of several cofactors (riboflavin, biotin, and cobalamin) in the shared OGs (Supplementary Table S7). The presence of these genes in “*Ca*. Nitrosocaldales/Nitrosocaldaceae”-core and other AOA-core sets confirms the conserved metabolic feature of AOA, which has been noted in previous study (Kerou et al., 2016). It was reported that the genomes of “*Ca.* Nitrosocaldus islandicus” and “*Ca.* Nitrosocaldus cavascurensis” encode a family B polymerase, and not family D polymerases (Abby et al., 2018; Daebeler et al., 2018). In our study, we found genes coding for PolB (OG0000200) in the shared set, together with a family Y DNA polymerase (OG0000329). Consistently, both subunits of PolD (OG0000966 and OG0001021) were detected in the “other AOA-accessory” set, but not in “*Ca.* Nitrosocaldales/Nitrosocaldaceae” (Supplementary Table S7 and Figure 2). PolB was therefore assumed to be the main replicative polymerase in “*Ca.* Nitrosocaldales/Nitrosocaldaceae,” as in most thermophilic Thermoproteales, also lack PolD (Barry and Bell, 2006). The absence of PolD in “*Ca.* Nitrosocaldales/Nitrosocaldaceae” indicates distinct mechanisms of DNA replication exist in “*Ca.* Nitrosocaldales/Nitrosocaldaceae” and other AOA. It might also be possible that DNA primases play a vital role, for example in lagging-strand synthesis, as several genes coding for DNA primases (OG0000234, OG0000417, and OG0000456) were detected in the shared set (Supplementary Table S7), and some archaeal primases were known to have polymerase activity (Lao-Sirieix and Bell, 2004).

The differences between the functional profiles of “*Ca*. Nitrosocaldales/Nitrosocaldaceae” and those of other AOA were relatively small but significant (Supplementary Figure S6, ADONIS *R^2^* = 0.11, *P* = 0.001). In total, 526 OGs were only present in “*Ca*. Nitrosocaldales/Nitrosocaldaceae,” which we therefore defined as “*Ca*. Nitrosocaldales/Nitrosocaldaceae”-specific OGs, and 5,410 OGs were only present in other AOA (other AOA-specific OGs). Among the 526 OGs, 43 of them could be classified as the core set of “*Ca*. Nitrosocaldales/Nitrosocaldaceae” (Supplementary Table S7). Notably, the gene coding for cyclic 2,3-diphosphoglycerate (cDPG) synthetase was found in this set, indicating that cDPG utilization might be a unique strategy used by “*Ca*. Nitrosocaldales/Nitrosocaldaceae” in hot springs (Abby et al., 2018) as it was reported that cDPG might be involved in the thermo-stabilization of proteins (Hensel and König, 1988; Shima et al., 1998). Additionally, cDPG could act as a storage reservoir for energy, phosphorus, or carbon. For example, cDPG could potentially help “*Ca*. Nitrosocaldales/Nitrosocaldaceae” survive during phosphate limitation, as reported in other archaea (Roberts, 2005). The detection of S-adenosylmethionine decarboxylase in this set suggests polyamine production, which was vital in cellular tolerance to high temperature (Tabor and Tabor, 1984; Morgan et al., 1987). Additionally, we predict that “*Ca*. Nitrosocaldales/Nitrosocaldaceae” can specifically export L-alanine to avoid the accumulation of amino acids to toxic levels (Kim et al., 2015), indicated by the presence of gene coding for L-alanine export (AlaE) in all “*Ca*. Nitrosocaldales/Nitrosocaldaceae” MAGs (Supplementary Tables S4, S7). The L-alanine could be generated in “*Ca.* Nitrosocaldales/Nitrosocaldaceae” MAGs (Supplementary Table S4) via cysteine desulfurase/selenocysteine lyase from selenocysteine or coupling alanine aminotransferase with glutamate dehydrogenase from pyruvate, as proposed in *Pyrococcus furiosus* (Kengen and Stams, 1994). However, alternative mechanisms of L-alanine accumulation might exist as the distribution of alanine aminotransferase was limited in “*Ca.* Nitrosocaldales/Nitrosocaldaceae” (totally absent in “*Ca.* Nitrosothermus”), which requires further study.

In terms of the “*Ca.* Nitrosocaldales/Nitrosocaldaceae”-accessory set, the largest proportion of them (34.9%) was related to defense mechanisms (Figure 5). Among them, genes of the CRISPR-Cas system, participating in resistance to viruses (Barrangou et al., 2007), were detected. Some of these genes have been associated with hyperthermophiles like Cas10, Cmr4g7, Cmr6g7, and Cmr1g7 (Makarova et al., 2003), indicating that these components of the CRISPR-Cas system might be necessary for managing viral infections in high-temperature ecosystems, where many archaeal viruses exist (Prangishvili, 2013; Gudbergsdóttir et al., 2016). Furthermore, a gene coding for indolepyruvate ferredoxin oxidoreductase was also included in the “*Ca.* Nitrosocaldales/Nitrosocaldaceae” accessory set, indicating the ability to ferment aromatic amino acids might be unique to “*Ca.* Nitrosocaldales/Nitrosocaldaceae.” Notably, the gene coding for cobalamin-dependent methionine synthase (MetH), a key enzyme of one-carbon metabolism, clustered with accessory set of other AOA whereas the cobalamin-independent methionine synthase (MetE) was present in “*Ca*. Nitrosocaldales/Nitrosocaldaceae.” It has been reported that MetH was more efficient than MetE (Helliwell et al., 2016), stressing the differences between “*Ca*. Nitrosocaldales/Nitrosocaldaceae” and other AOA.

In summary, the comparisons of function profiles between “*Ca*. Nitrosocaldales/Nitrosocaldaceae” and other AOA could identify potential mechanisms of thermophilic adaptation in “*Ca*. Nitrosocaldales/Nitrosocaldaceae.”

### The Role of HGT in “*Ca.* Nitrosocaldaceae”

Previous studies have stressed the role of HGT in the adaptation of Thaumarchaeota (López-García et al., 2015; Herbold et al., 2017; Ren et al., 2019). However, little is known how HGT events have affected “*Ca.* Nitrosocaldales/Nitrosocaldaceae.” To probe the effect of HGT in adaptations of “*Ca.* Nitrosocaldales/Nitrosocaldaceae,” initial analysis was conducted assisted by an automated pipeline HGTector2 (Zhu et al., 2014) to identify candidate HGT genes and some were subsequently confirmed by phylogenetic analyses. In our study, more than 8% of “*Ca.* Nitrosocaldales/Nitrosocaldaceae” genes might have been horizontally transferred, and as typical, few candidate HGTs were involved in information processing (Supplementary Figure S7A). Among all candidate HGTs, genes related to carbohydrate metabolism (∼10.55% of the total candidate HGTs), amino acid metabolism (∼10.47%), energy metabolism (∼8.53%), membrane transport (∼6.51%), and metabolism of cofactors and vitamins (6.33%), were the top five most abundant functional classifications (Supplementary Figure S7B). Among the potential donors, more genes were acquired from archaea (505), with Crenarchaeota transferring the largest number (146). Nevertheless, the contribution of bacteria (422) was significant, including many genes (47) that were acquired from Firmicutes (Supplementary Figure S8).

In the present study, it was noticed that all copies of the SOD genes, encoding for superoxide dismutase required for detoxification of reactive oxygen species (ROS), were potentially horizontally transferred. ROS would be produced during ammonia oxidation (Kim et al., 2016) and aerobic respiration. Most of these SOD might have been transferred from Crenarchaeota or Euryarchaeota (Supplementary Figure S9). Other essential HGT genes necessary for protection from ROS include *speE*, and *trxB*. Among them, *trxB*, encoding for thioredoxin reductase, could help repair oxidatively damaged cytoplasmic proteins and protect cells from oxidative stress (Cheng et al., 2017). Spermidine synthase, encoded by gene *speE*, could provide DNA protection and stability in thermal habitats (Cheng et al., 2009). These genes would enable “*Ca.* Nitrosocaldales/Nitrosocaldaceae” to broaden their niche into low-oxygen or anoxic conditions. Gene *pspA* was included among the HGT candidate, which encodes phage shock protein A involved in response to several stresses (heat, ethanol, and osmotic shock) (Brissette et al., 1990; Kleerebezem et al., 1996). Phylogenetic analysis showed that they might have been transferred from Euryarchaeota (Supplementary Figure S10). Genes encoding for ATP-dependent helicase Lhr and Lhr-like helicase might have transferred from other archaea. These genes have been reported to play an important role in DNA repair (Rand et al., 2003). Other HGTs from archaea that might be important in coping with stress included genes coding for multiple antibiotic resistance protein imported from Bathyarchaeota and genes coding for Type I restriction enzyme proteins that protect cells from viral infection (Ahlgren et al., 2017) imported from Euryarchaeota. All the above results revealed that archaeal HGT genes contributed largely to stress responses in “*Ca.* Nitrosocaldales/Nitrosocaldaceae.” Gene *arsC* encoding for arsenate reductase, involved in detoxification of arsenate (Rosen and Liu, 2009), was imported from bacteria (Supplementary Figure S11).

Some genes involved in the CRISPR-Cas system might belong to HGT genes, which assist “*Ca.* Nitrosocaldales/Nitrosocaldaceae” to defend against virus infection. In addition, “*Ca.* Nitrosocaldales/Nitrosocaldaceae” acquired several genes coding for proteins involved in phosphate transport from bacteria, including permease protein, ATP-binding protein, and substrate-binding protein, which likely enable “*Ca.* Nitrosocaldales/Nitrosocaldaceae” to take up phosphate in low and/or fluctuating phosphate concentrations. Detailed information on HGT genes is shown in Supplementary Table S8.

Frequent HGTs have previously been identified in thermophiles, facilitating their adaptation in high-temperature habitats (Aravind et al., 1998; Rhodes et al., 2011). Our study showed that “*Ca.* Nitrosocaldaceae” might have acquired genes from both domains, which may be an essential driver to allow this family to adapt to phosphate-limited and thermal habitats but the confirmation necessitates further studies.

## Conclusion

In the present study, a total of nine MAGs belonging to “*Ca.* Nitrosocaldales/Nitrosocaldaceae” from India and China were recovered from hot spring sediments, which enabled us to obtain a better picture of phylogenetic diversity and metabolic potential of this family. In all, we showed that the “*Ca.* Nitrosocaldales/Nitrosocaldaceae” belong to four clades and propose two new species of “*Ca.* Nitrosocaldus” and one new genus, “*Ca.* Nitrosothermus,” to accommodate the new genomes. Similar to other AOA, the potential for ammonia oxidation and carbon fixation via thaumarchaeal 3HP/4HB pathway was conserved in all “*Ca.* Nitrosocaldaceae” MAGs but urea utilization and biosynthesis of vitamins, biotin, cobalamin, and riboflavin were detected in some MAGs. The potential adaptive features of “*Ca*. Nitrosocaldales/Nitrosocaldaceae” were explored in this study. AOA shared many conserved genes, and their central metabolism was highly conserved (ammonia oxidation and carbon fixation). However, specific features exist in “*Ca*, Nitrosocaldales/Nitrosocaldaceae” (e.g., cDPG synthesis), stressing their adaptation mechanisms. It was found that >8% (from bacteria and archaea) of the genes in “*Ca.* Nitrosocaldaceae” might be horizontally transferred, and the majority of them were acquired from the same domain, suggesting that HGT played an important role in adapting to thermal habitats. In summary, our study gives an insight into the metabolic potentials and possible adaptations of “*Ca.* Nitrosocaldaceae” in thermal habitats, which shed light on the elucidation of the adaptation mechanisms enabling the ecological success of AOA in different environments.

## Data Availability Statement

The datasets generated for this study can be found in the online repositories. The names of the repository/repositories and accession number(s) can be found in the article/Supplementary Material.

## Author Contributions

Z-HL, MPNR, and BH wrote the manuscript. HC, Z-SH, QL, Z-YD, B-BL, and S-XG performed the metagenomic analysis, genome binning, functional annotation, and evolutionary analysis. W-SS and W-JL guided the project. All authors discussed the results and commented on the manuscript.

## Conflict of Interest

The authors declare that the research was conducted in the absence of any commercial or financial relationships that could be construed as a potential conflict of interest.

## Footnotes

1 http://sourceforge.net/projects/bbmap/

2 https://www.ncbi.nlm.nih.gov/

3 https://img.jgi.doe.gov/cgi-bin/m/main.cgi

4 https://www.genome.jp/kegg/kegg1b.html

5 http://sankeymatic.com/

6 https://github.com/dparks1134/CompareM
