# Supplementary material for: Genomic Insights of “Candidatus Nitrosocaldaceae” Based on Nine New Metagenome-Assembled Genomes, Including “Candidatus Nitrosothermus” Gen Nov. and Two New Species of “Candidatus Nitrosocaldus”
Source: Front Microbiol. 2021 Jan 8;11:608832. doi: 10.3389/fmicb.2020.608832 (PMC7819960; doi:10.3389/fmicb.2020.608832)
Supplement: Supplementary file 2 [file Data_Sheet_2.docx]

**Genomic insights of *“Candidatus* Nitrosocaldaceae” based on nine new metagenome-assembled genomes, including “*Candidatus* Nitrosothermus” gen nov. and two new species of “*Candidatus* Nitrosocaldus”**

Zhen-Hao Luo^1^, Manik Prabhu Narsing Rao^1^, Hao Chen^1^, Zheng-Shuang Hua^1,3^, Qi Li^1^, Brian P. Hedlund ^4,5^, Zhou-Yan Dong^1^, Bing-Bing Liu^2^, Shu-Xian Guo^2^, Wen-Sheng Shu^6*^, Wen-Jun Li^1,2*^

^1^State Key Laboratory of Biocontrol, Guangdong Provincial Key Laboratory of Plant Resources, School of Life Sciences, Sun Yat-Sen University, Guangzhou, 510275, PR China

^2^Henan Key Laboratory of Industrial Microbial Resources and Fermentation Technology, College of Biological and Chemical Engineering, Nanyang Institute of Technology, Nanyang 473004, PR China

^3^Department of Biological Sciences, Dartmouth College, Hanover, NH 03755, USA

^4^School of Life Sciences, University of Nevada Las Vegas, Las Vegas, NV 89154, USA.

^5^Nevada Institute of Personalized Medicine, University of Nevada Las Vegas, Las Vegas, NV 89154, USA

^6^School of Life Sciences, South China Normal University, 510631 Guangzhou, PR China

^*^ Correspondence authors: W.J.L., liwenjun3@mail.sysu.edu.cn

W.S.S, shuwensheng@m.scnu.edu.cn

**
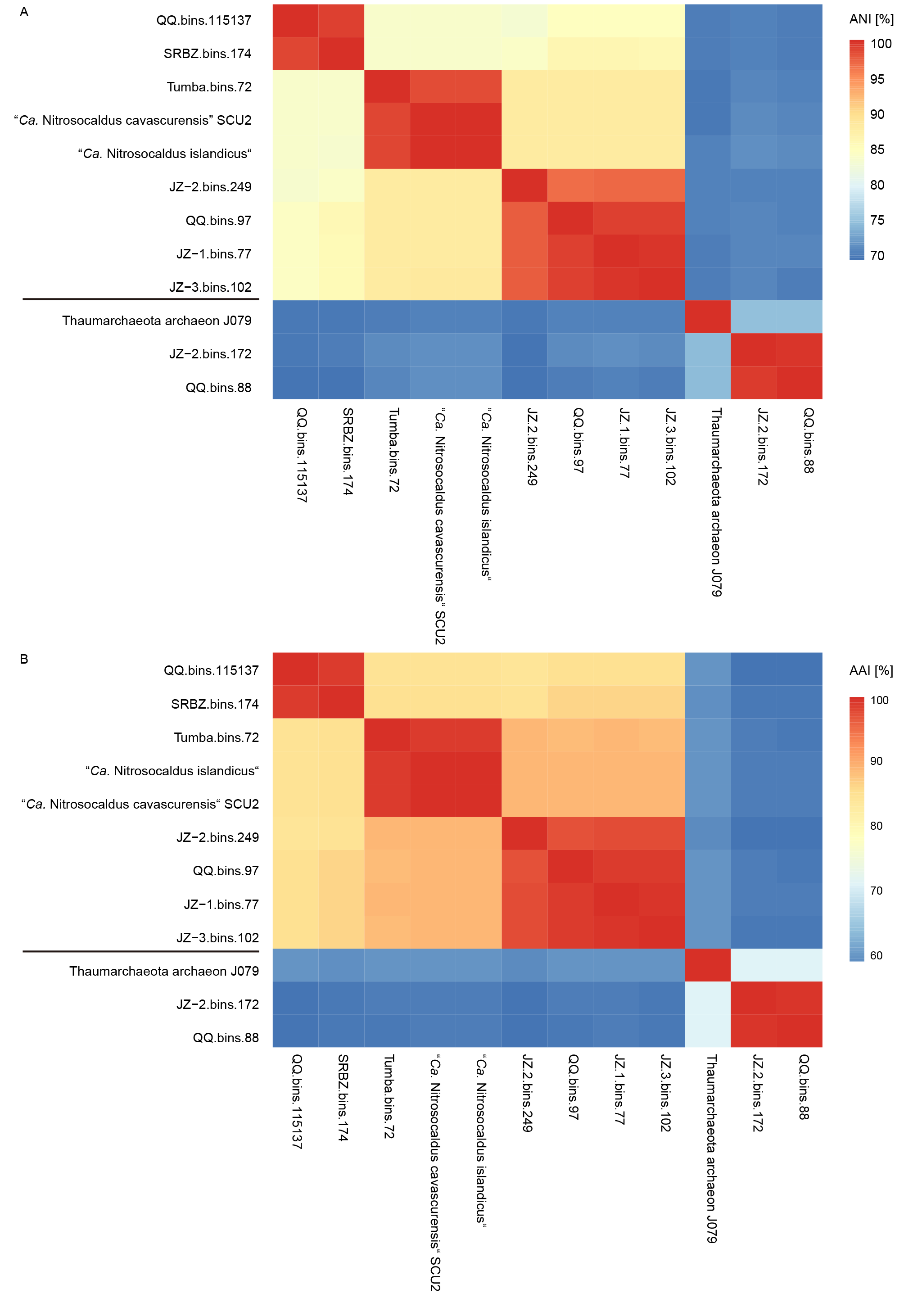
**

**FIGURE S1| (A)** Average amino acid identity (AAI) and **(B)** average nucleotide identity (ANI) of 12 MAGs of ***“Ca.* Nitrosocaldaceae”**. Genus level delineation are marked by black lines.


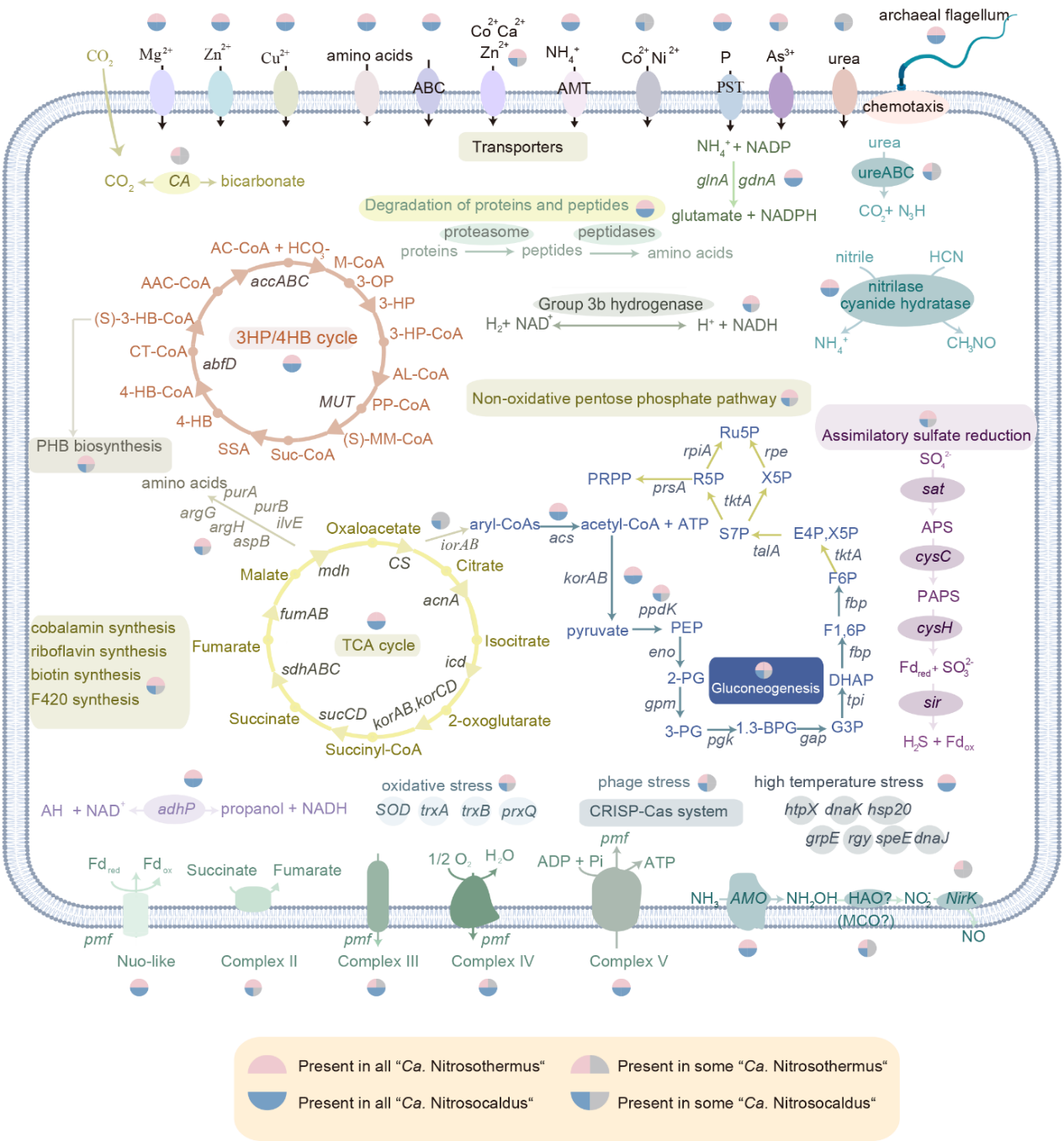


**FIGURE S2|** Reconstructed metabolic pathways of ***“Ca.* Nitrosocaldaceae”**. AAC-CoA, acetoacetyl-CoA; AC-CoA, acetyl-CoA; AH, aldehyde; AL-CoA, acryloyl-CoA; AMO, ammonia monooxygenase; APS, adenylyl sulfate; 1,3-BPG, glycerate-1,3P_2_; CA, carbonic anhydrase; CT-CoA, crotonoyl-CoA; DHAP, glycerine-P; E4P, D-erythrose-4P; Fdox, oxidized ferredoxin; Fdred, reduced ferredoxin; F6P, β-D-fructose-6P; F1,6P, β-D-fructose-1,6P_2_; G3P, glyceraldehyde-3P; HAO, hydroxylamine dehydrogenase; 4-HB-CoA, 4-hydroxybutyryl-CoA; 4-HB, 4-hydroxybutanoic acid; HCN, hydrogen cyanide; 3-HP, 3-hydroxypropanoate; 3-HP-CoA, 3-hydroxypropionyl-CoA; 3HP/4HB, 3-hydroxypropionate/4-hydroxybutyrate; MCO, multicopper oxidase; M-CoA, malonyl-CoA; NirK, nitrite reductase (NO-forming); 3-OP, 3-oxopropanoate; PAPS, 3’-phosphoadenylyl sulfate; PEP, phosphoenolpyruvate; 2-PG, glycerate-2P; 3-PG, glycerate-3P; PHB, poly-β-hydroxybutyrate; pmf, proton motive force; PP-CoA, propenoyl-CoA; PRPP, 5-Phospho-alpha-D-ribose 1-diphosphate; R5P, D-ribose-5P; Ru5P, D-ribulose-5P; (S)-3-HB-CoA, (S)-3-hydroxybutanoyl-CoA; (S)-MM-CoA, (S)-methylmalonyl-CoA; S7P, D-Sedo-heptulose-7P; SSA, succinate semialdehyde; Suc-CoA, succinyl-CoA. For detailed on pathways see Table S4.


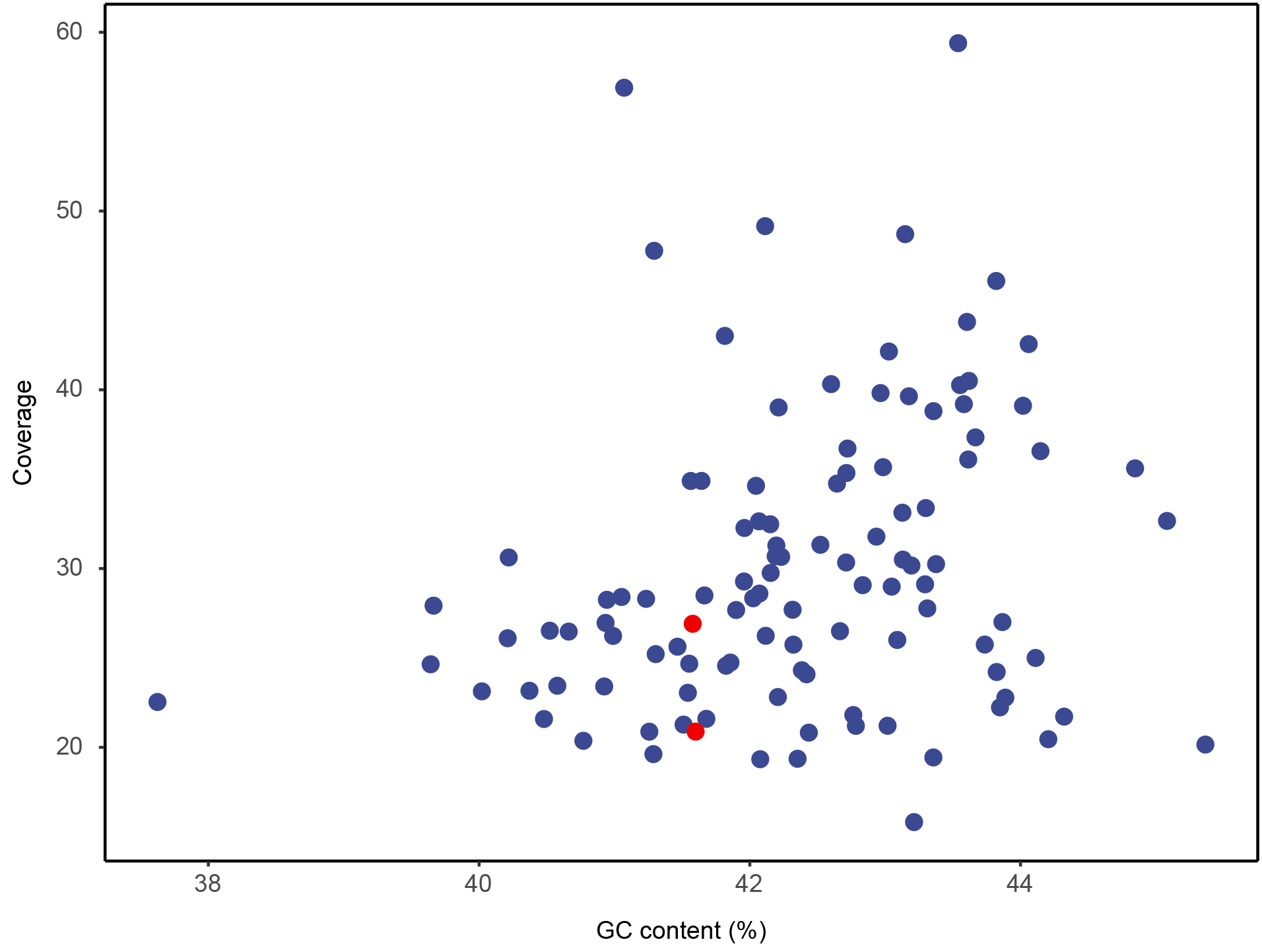


**FIGURE S3|** GC content and coverage plot of all scaffolds in JZ-2.bins.249. Scaffolds with *amoA* gene are marked with red while others are with blue.


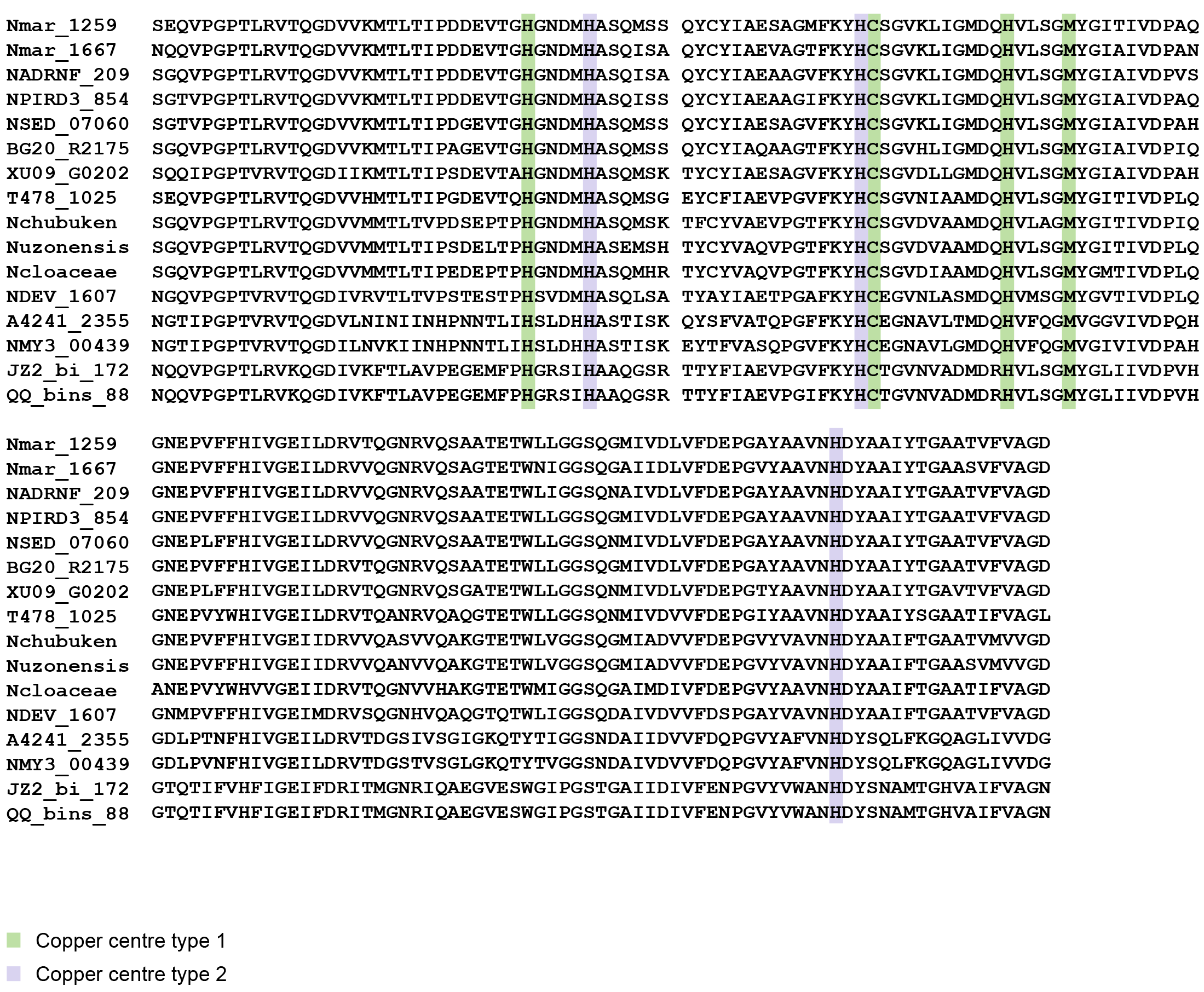


**FIGURE S4|** Alignments of nitrite reductase (NirK) from sequences in the dashed box in Fig S7. Copper coordinating residues are highlighted with green and purple indicating copper centre type 1 and type 2, inferred from Bartossek et al., 2010. Nitrite reductases are from *Nitrosopumilus maritimus* SCM1 (Nmar_1259 and Nmar_1667), *Nitrosopumilus adriaticus* (NADRNF_209), *Nitrosopumilus piranensis* (NPIRD3_854), “*Ca.* Nitrosopumilus sediminis” (NSED_07060), “*Ca.* Nitrosarchaeum limnium” SFB1 (BG20_R2175), Thaumarchaeota archaeon CSP1-1 (XU09_G0202), “*Ca*. Nitrosopelagicus brevis” (T478_1025), “*Ca.* Nitrosotenuis chungbukensis” (Nchubuken), “*Ca.* Nitrosotenuis uzonensis” (Nuzonensis), “*Ca.* Nitrosotenuis cloaceae” (Ncloaceae), “*Ca.* Nitrosotalea devanaterra” Nd1 (NDEV_1607), “*Ca.* Nitrosocosmucis exaquare” G61 (A4241_2355), “*Ca.* Nitrosocosmicus oleophilus” MY3 (NMY_00439), JZ-2.bins.172 (JZ2_bi_172), and QQ.bins.88 (QQ_bins_88).


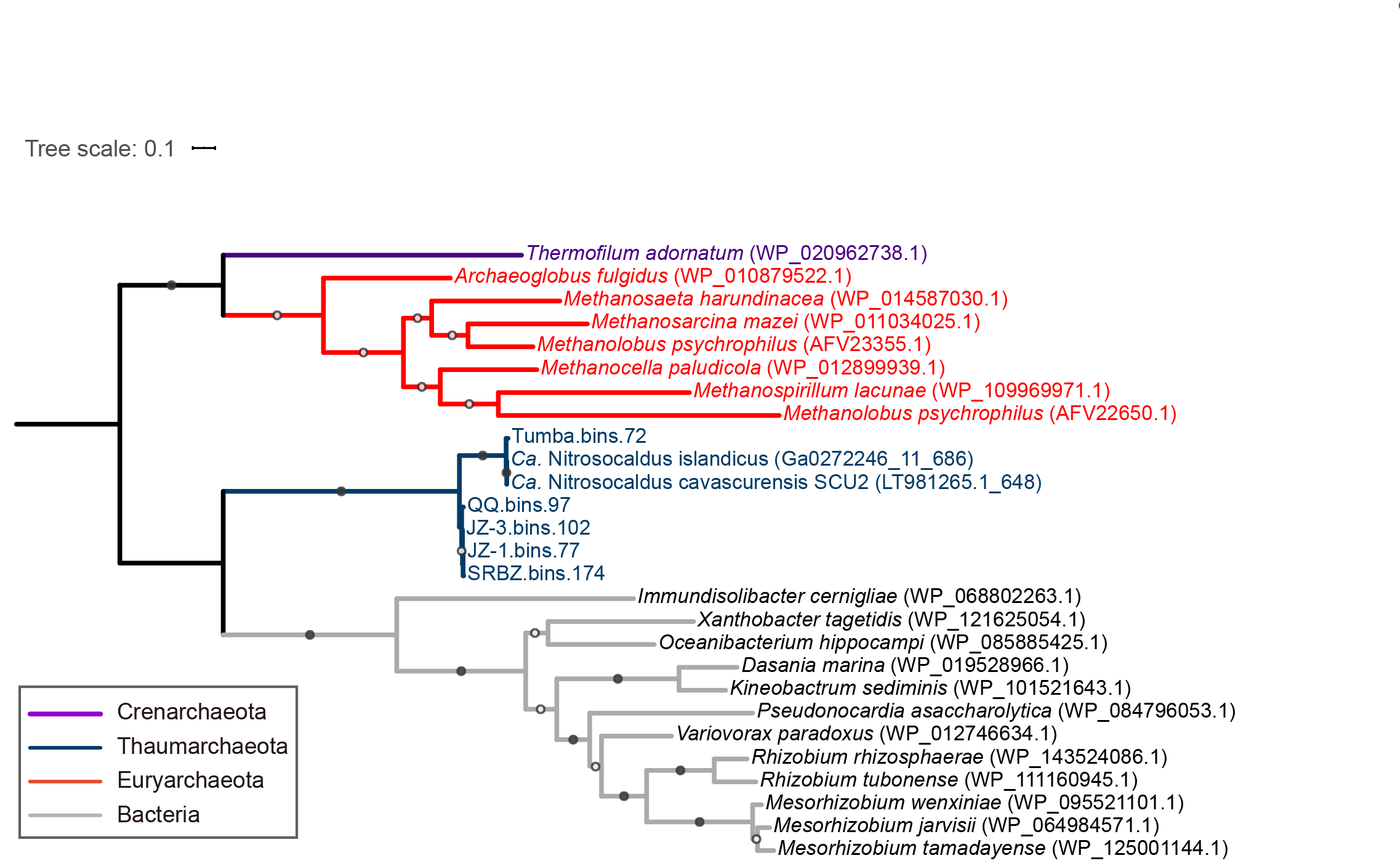


**FIGURE S5|** Phylogenetic tree of beta subunit of indolepyruvate ferredoxin oxidoreductase (IorB). Sequences from different phylum (domains) are marked with different colors. Nodes with ultrafast bootstrap values are indicated as solid circles (≥ 95%) and hollow circles (≥50% and < 95%), and the scale bar at the top indicates 10% sequence divergence.


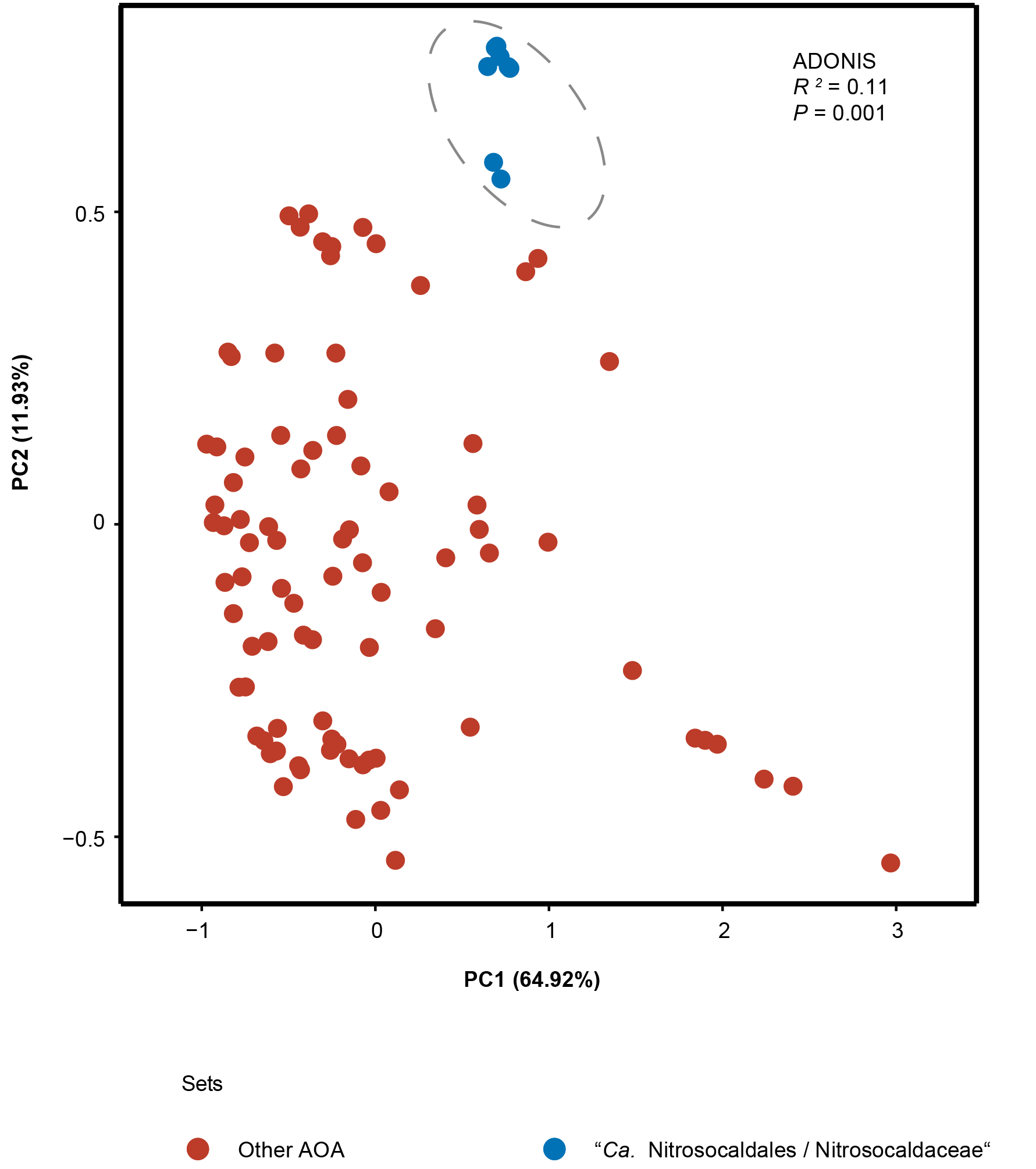


**FIGURE S6|** Principal coordination analysis (PCoA) plot with Bray-Curtis dissimilatory based on OG profiles of all selected AOA genomes. The analysis of similarity (ADONIS) statistics considers genomes between “*Ca.* Nitrosocaldales/Nitrosocaldaceae” and other AOA (inside and outside the dashed ellipses).


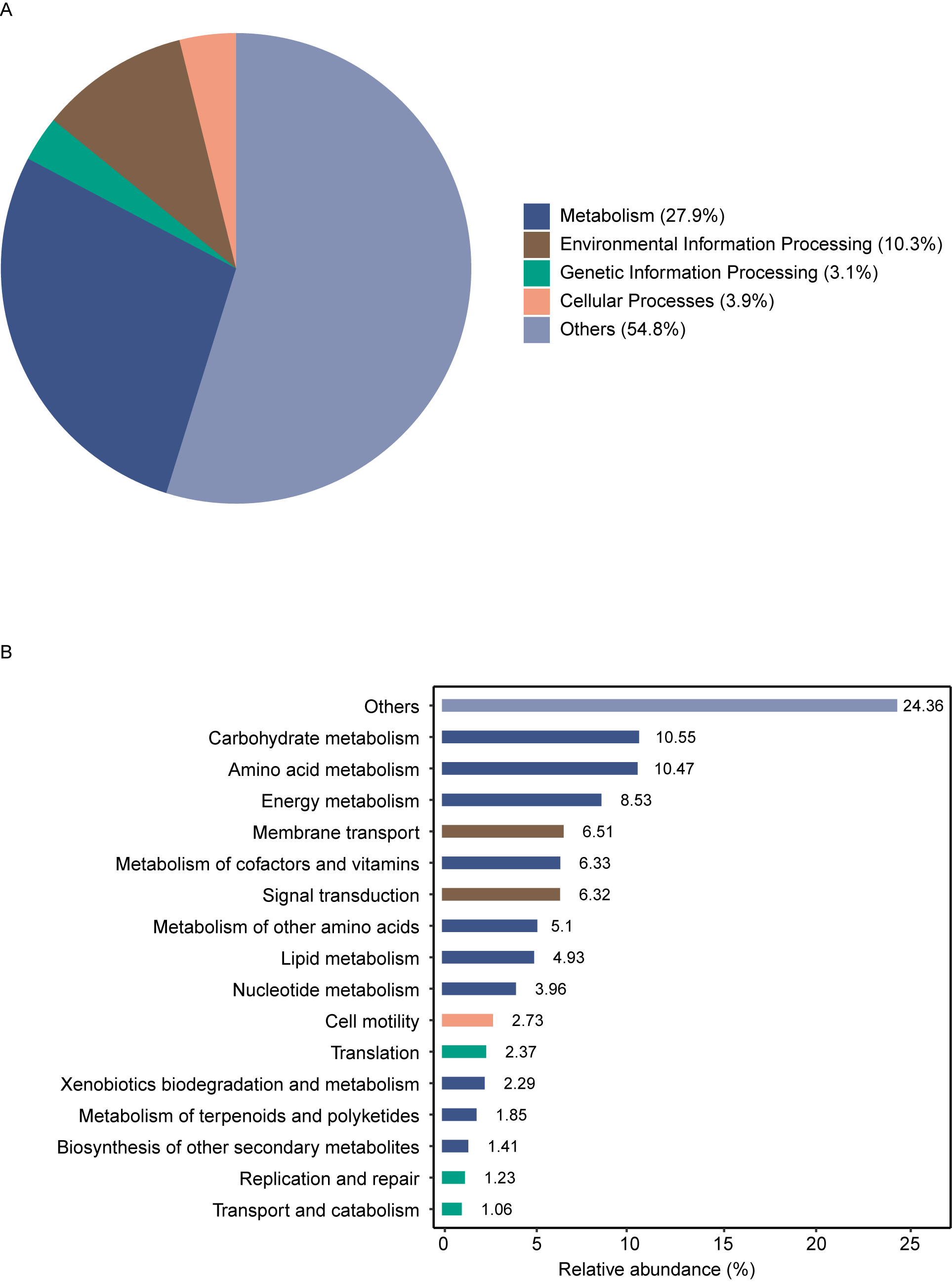


**FIGURE S7| (A)** Proportions of each metabolic category (Level 1) of identified HGTs based on KEGG annotation result. **(B)** Relative abundances of each metabolic category (Level 2) of identified HGTs based on KEGG annotation result. The definition of colors is according to categories as displayed in A.

**
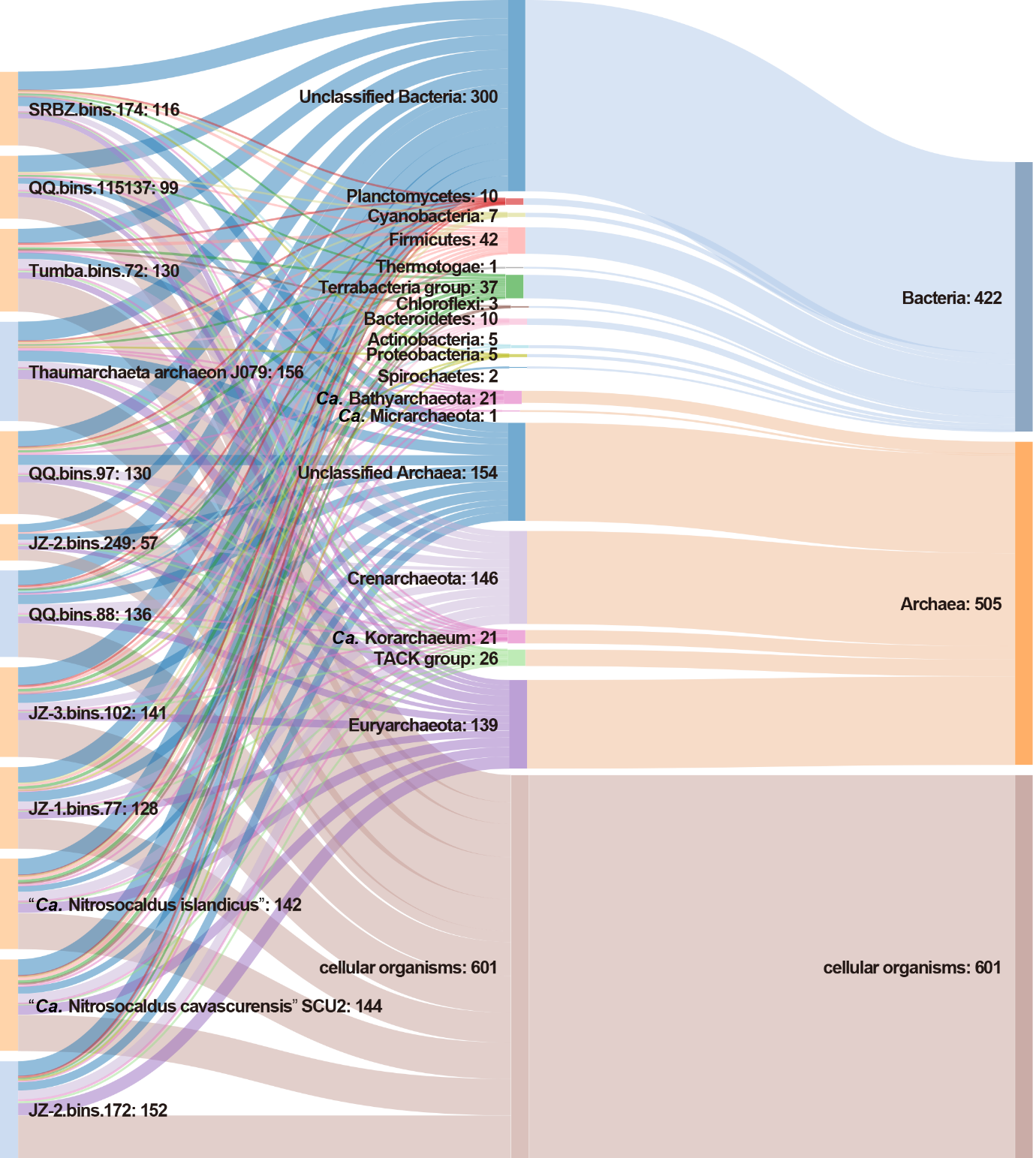
**

**FIGURE S8|** The Sankey plot of the detected HGTs and potential donors for each genome, generated using SankeyMATIC (sankeymatic.com/).


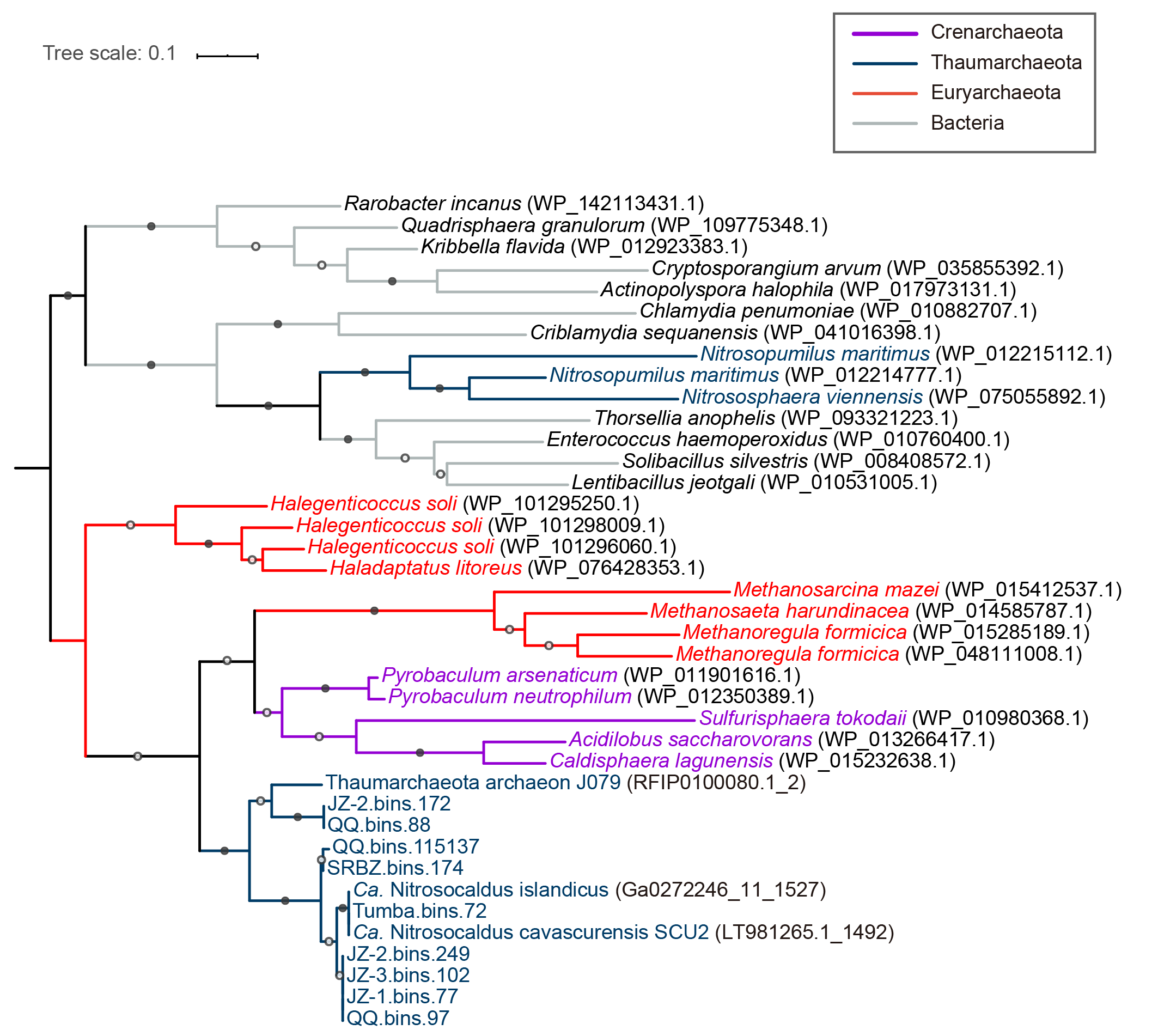


**FIGURE S9|** Phylogenetic tree of superoxide dismutase, Fe-Mn family (SOD). Sequences from different phylum (domains) are marked with different colors. Nodes with ultrafast bootstrap values are indicated as solid circles (≥ 95%) and hollow circles (≥50% and < 95%), and the scale bar at the top indicates 10% sequence divergence.


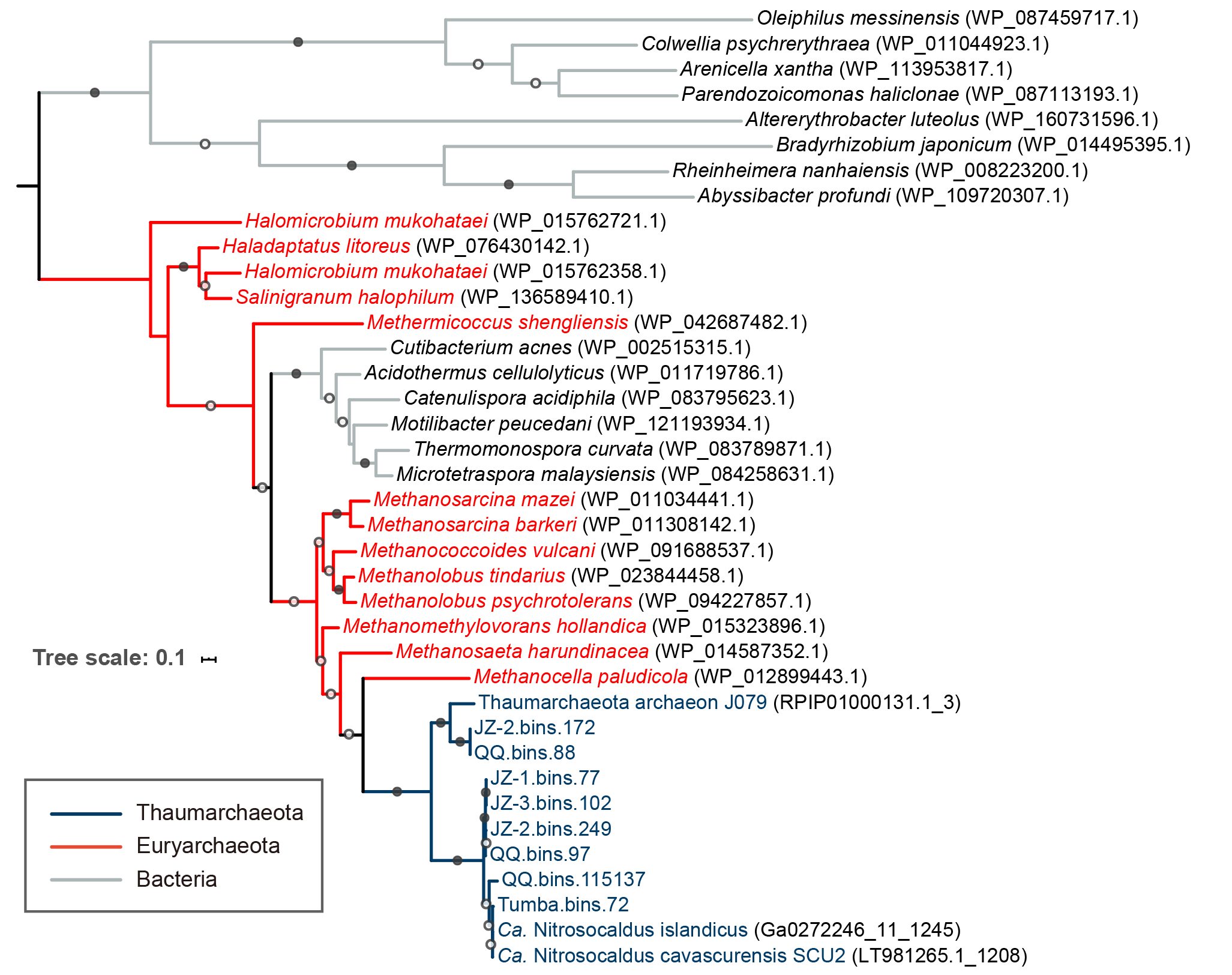


**FIGURE S10|** Phylogenetic tree of phage shock protein A (PspA). Sequences from different phylum (domains) are marked with different colors. Nodes with ultrafast bootstrap values are indicated as solid circles (≥ 95%) and hollow circles (≥50% and < 95%), and the scale bar at the bottom indicates 10% sequence divergence.


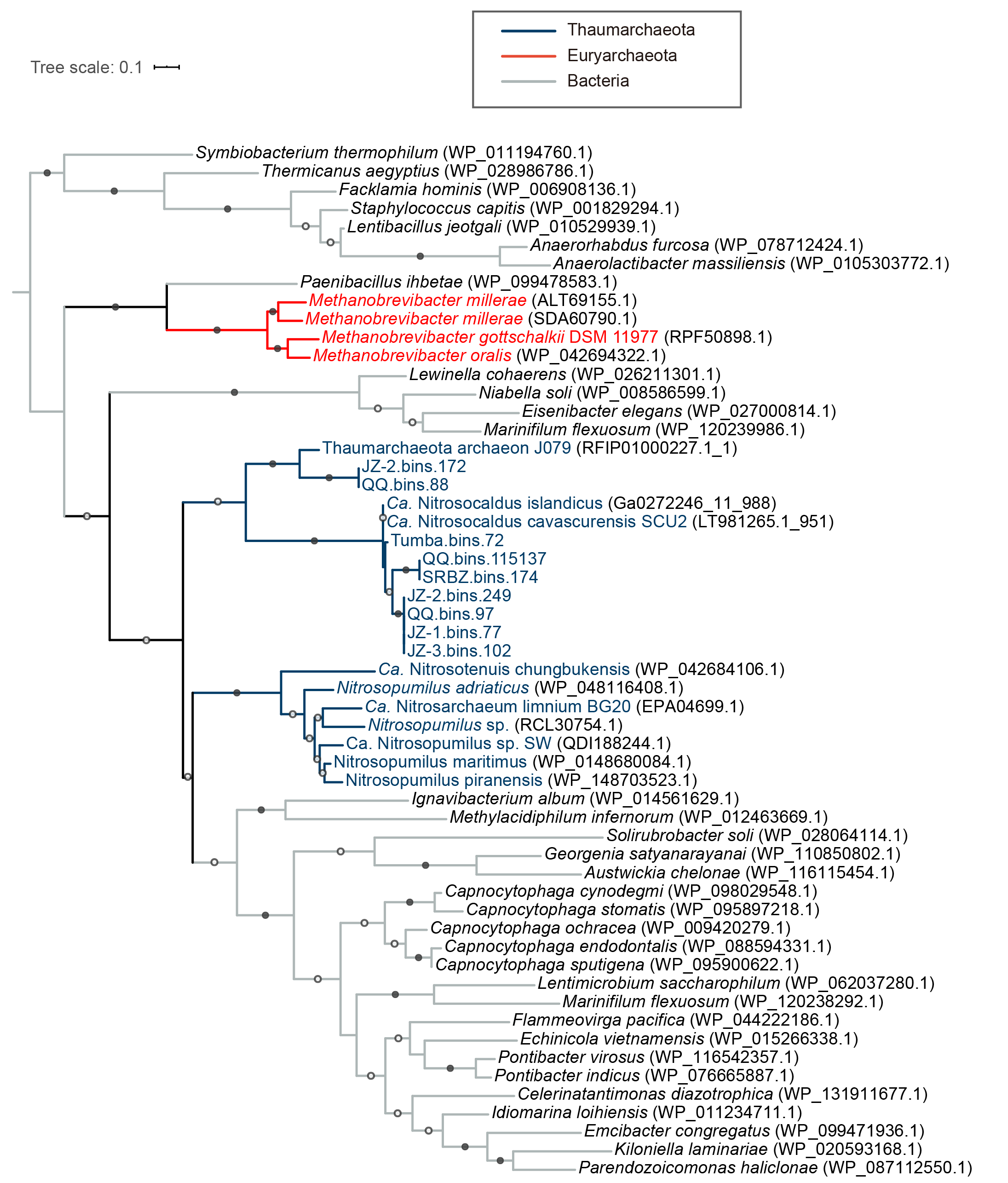


**FIGURE S11|** Phylogenetic tree of arsenate reductase (glutaredoxin) (ArsC). Sequences from different phylum (domains) are marked with different colors. Nodes with ultrafast bootstrap values are indicated as solid circles (≥ 95%) and hollow circles (≥50% and < 95%), and the scale bar at the top indicates 10% sequence divergence.

**References**

Bartossek, R., Nicol, G. W., Lanzen, A., Klenk, H. P., and Schleper, C. (2010). Homologues of nitrite reductases in ammonia-oxidizing archaea: diversity and genomic context. *Environ. Microbiol*. 12, 1075–1088. doi: 10.1111/j.1462-2920.2010.02153.x
